# ^129^Xe Image Processing Pipeline: An open‐source, graphical user interface application for the analysis of hyperpolarized ^129^Xe MRI

**DOI:** 10.1002/mrm.30347

**Published:** 2024-10-31

**Authors:** Abdullah S. Bdaiwi, Matthew M. Willmering, Joseph W. Plummer, Riaz Hussain, David J. Roach, Juan Parra‐Robles, Peter J. Niedbalski, Jason C. Woods, Laura L. Walkup, Zackary I. Cleveland

**Affiliations:** ^1^ Center for Pulmonary Imaging Research, Division of Pulmonary Medicine Cincinnati Children's Hospital Medical Center Cincinnati Ohio USA; ^2^ Department of Biomedical Engineering University of Cincinnati Cincinnati Ohio USA; ^3^ Division of Pulmonary, Critical Care, and Sleep Medicine, Department of Internal Medicine University of Kansas Medical Center Kansas City Kansas USA; ^4^ Department of Bioengineering University of Kansas Lawrence Kansas USA; ^5^ Hoglund Biomedical Imaging Center University of Kansas Medical Center Kansas City Kansas USA; ^6^ Department of Pediatrics University of Cincinnati Cincinnati Ohio USA; ^7^ Imaging Research Center, Department of Radiology Cincinnati Children's Hospital Medical Center Cincinnati Ohio USA

**Keywords:** diffusion analysis, gas exchange analysis, hyperpolarized ^129^Xe, ventilation analysis

## Abstract

**Purpose:**

Hyperpolarized ^129^Xe MRI presents opportunities to assess regional pulmonary microstructure and function. Ongoing advancements in hardware, sequences, and image processing have helped it become increasingly adopted for both research and clinical use. As the number of applications and users increase, standardization becomes crucial. To that end, this study developed an executable, open‐source ^129^Xe image processing pipeline (XIPline) to provide a user‐friendly, graphical user interface‐based analysis pipeline to analyze and visualize ^129^Xe MR data, including scanner calibration, ventilation, diffusion‐weighted, and gas exchange images.

**Methods:**

The customizable XIPline is designed in MATLAB to analyze data from all three major scanner platforms. Calibration data is processed to calculate optimal flip angle and determine^129^Xe frequency offset. Data processing includes loading, reconstructing, registering, segmenting, and post‐processing images. Ventilation analysis incorporates three common algorithms to calculate ventilation defect percentage and novel techniques to assess defect distribution and ventilation texture. Diffusion analysis features ADC mapping, modified linear binning to account for ADC age‐dependence, and common diffusion morphometry methods. Gas exchange processing uses a generalized linear binning for data acquired using 1‐point Dixon imaging.

**Results:**

The XIPline workflow is demonstrated using analysis from representative calibration, ventilation, diffusion, and gas exchange data.

**Conclusion:**

The application will reduce redundant effort when implementing new techniques across research sites by providing an open‐source framework for developers. In its current form, it offers a robust and adaptable platform for ^129^Xe MRI analysis to ensure methodological consistency, transparency, and support for collaborative research across multiple sites and MRI manufacturers.

## 1 INTRODUCTION

Hyperpolarized (HP) ^129^Xe MRI has emerged as a powerful pulmonary imaging technique that offers high signal and unique contrast without ionizing radiation.^1^, ^2^ This enables regional ventilation, alveolar‐airspace microstructure, and gas exchange to be visualized and quantified within the lungs. Because of its excellent safety profile,^3^, ^4^ sensitivity to early pathology and high reproducibility,^5^, ^6^, ^7^ ^129^Xe MRI is ideally suited to assess lung pathology longitudinally.^8^ Recent advances in coil hardware,^3^, ^4^ sequence design,^5^, ^6^, ^9^ and image reconstruction^10^, ^11^, ^12^, ^13^, ^14^ are expected to further increase the use of HP ^129^Xe MRI in human subjects. Moreover, ^129^Xe MRI has transitioned from basic and translational science to clinical implementation, with the University of Sheffield in the United Kingdom receiving approval from the Medicines and Healthcare products Regulatory Agency to clinically produce HP gases in 2015. Following, approval over 500 patients have been referred for HP ^129^Xe MRI through the United Kingdom National Health Service.^15^ In the United States (US), phase‐III clinical trials were successfully concluded in January 2020, and the US Food and Drug Administration (FDA) approved ^129^Xe MRI for assessing ventilation in patients 12 years of age and older in late 2022.^16^, ^17^ As the number of sites able to perform routine clinical and translational HP ^129^Xe MRI grow, the technology will become increasingly well‐positioned to serve as an endpoint in multi‐site clinical trials that assess the efficacy of new therapies for lung diseases.

To enable robust and routine multi‐site trials with HP ^129^Xe MRI, data acquisition and analysis must be standardized. Therefore, it is crucial to establish protocols and methods that can be made uniform across sites and implemented easily—particularly for sites that lack extensive expertise with HP gas MRI.^18^ To facilitate the use HP ^129^Xe MRI in multi‐site translational and clinical research, the ^129^Xe MRI Clinical Trials Consortium (XeCTC) (https://www.129xectc.org/) recently published a position paper describing harmonized and easily implemented ^129^Xe MRI acquisition protocols.^15^ However, standardized protocols must still be developed to enable robust and harmonized analysis of multi‐site data.

Challenges that hinder standardized data processing across sites include the (1) lack of automated center frequency determination of ^129^Xe data (particularly for non‐Cartesian data), (2) the absence of online flip‐angle calibration for non‐equilibrium X‐nucleus imaging, and (3) platform and vendor‐specific raw data structures and image reconstruction conventions. Further, the large and often flexible coils used in ^129^Xe MRI typically display significant B_1_ inhomogeneity, which necessitates bias‐field correction.^19^, ^20^, ^21^ These challenges have historically encouraged the use of site‐specific mixtures of on‐line and custom off‐line image reconstruction, correction, and analysis tools, which introduce another—arguably more significant—layer of complexity when comparing results across study sites. These challenges must be addressed, and imaging pipelines must be systematically harmonized, to generate the precise and reliable data needed for robust, multi‐site clinical studies.

To address the need for standardized data processing and analysis, we developed ^129^Xe image processing pipeline (XIPline). XIPline is an executable application in MATLAB R2023b (The MathWorks) that provides a user‐friendly, graphical user interface (GUI)‐based pipeline to import, reconstruct (if needed), process, and analyze HP ^129^Xe MR data. By incorporating a GUI, the analysis process simplified and made more accessible to researchers with varying levels of image processing and coding expertise. Moreover, the application is designed to be highly modular, allowing new analysis methods and functions to be incorporated as technology, sequences, and clinical‐translational applications evolve. Finally, the open‐source framework will promote transparency, community involvement, and collaboration across the growing HP ^129^Xe MRI community.

## 2 METHODS

### 2.1 Application overview and layout

**FIGURE 1:**
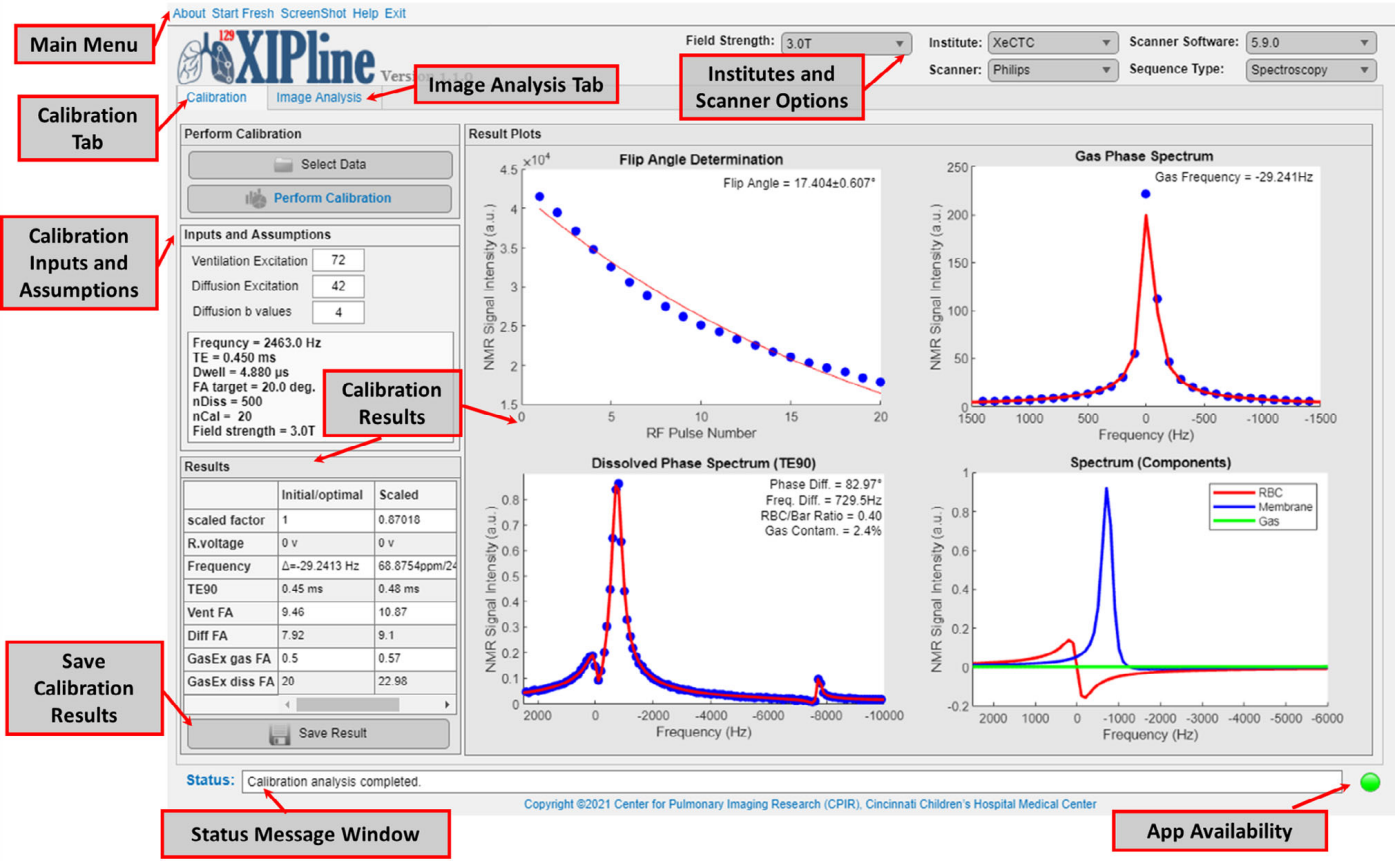
^129^Xe image processing pipeline (XIPline) and “Calibration” tab layout. A brief XIPline description and help documents are found in the main menu. Institutes and scanner options allow for site‐specific customization. Two key tabs drive the workflow: the Calibration tab is used to analyze data from a dedicated calibration scan, whereas the “Image Analysis” tab enables processing and analysis of ^129^Xe images. A status window with a text box provides real‐time task updates and error messages.

XIPline was developed using MATLAB R2023b with the Application Designer Toolbox. Figure 1 illustrates the fundamental layout of the compiled application. Notably, the institution and scanner options (top right) offer the ability to customize the application for meeting the site‐specific requirements, which will be particularly valuable when working across multiple scanners (vendors, hardware generations, field strengths, etc.), different scanner software versions, and classes of pulse sequence. Note, the “Institution” option in XIPline provides developers and advanced users with the flexibility to implement custom workflows or institution‐specific analysis protocols, while maintaining the core functionality of the application for general use. XIPline is structured into two primary tabs. The “Calibration” tab is dedicated to analyzing data from a dedicated spectroscopy‐based calibration scan. It computes operating parameters such as flip angles and ^129^Xe center frequency for subsequent Xe ventilation, diffusion‐weighted, or gas‐exchange acquisitions. The status window, featuring a text box, offers real‐time updates on ongoing tasks and displays error messages when necessary.

**FIGURE 2:**
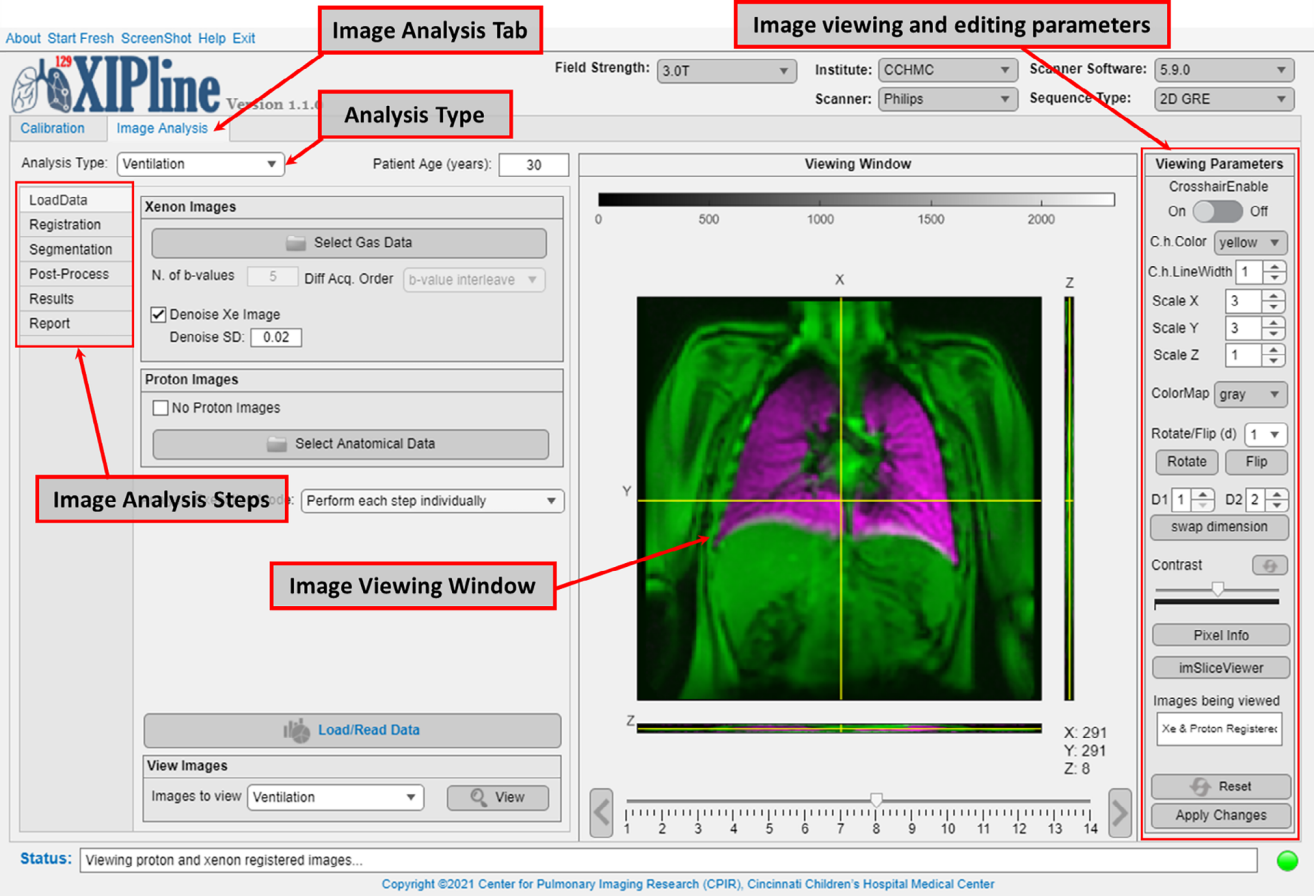
^129^Xe image processing pipeline and “Image Analysis” tab layout. This application guides users through ^129^Xe MRI analysis for ventilation, diffusion, and gas exchange. After selecting the analysis type, users load ^129^Xe and anatomical data (raw or reconstructed) (Figure S1, Table 2). If available, anatomical images can be registered with ^129^Xe images for improved lung segmentation (Figure S2). Lung masks are then created using manual tools, thresholding, or pretrained models (Figure S3). Analysis settings and methods are chosen in the “Post‐Processing” panel for ventilation (Figure S4), diffusion (Figure S5), and gas exchange (Figure S6). Results summaries and visualizations are displayed in the “Results” panel, with patient reports generated under the Report panel. The right‐side window allows image visualization with basic editing tools.

Figure 2 shows the layout of the “Image Analysis” tab, which enables processing and analyzing of ^129^Xe images. This includes first importing images reconstructed on‐line or raw data for off‐line image reconstruction (Figures S1). Reconstructed images can then be registered (e.g., with corresponding ^1^H anatomical images) (Figures S2), segmented to generate lung masks (Figure S3), and analyzed using various post‐processing routines. The “Post‐Process” panel (Figures S4–S6) further encompasses three additional tabs, each containing analysis input parameters for quantifying ventilation, diffusion, or gas‐exchange images. Similarly, under the “Results” panel, three separate tabs display the primary findings for each analysis type. Moreover, XIPline includes a report panel that can be used to generate and display subject‐specific analyses (e.g., subject info, scan info, summary of findings, etc., see Figure S7). The Image Analysis tab also provides a display window and basic image processing tools (e.g., flipping, rotation, etc) to visualize and manipulate the data.

### 2.2 Calibration analysis

For conventional proton (^1^H) MRI, important image acquisition parameters—including transmitter power, excitation frequency, and receiver frequency—are typically calibrated using automated routines offered by vendors. Unfortunately, automated calibration routines for multi‐nuclear (^129^Xe) acquisitions are typically not available. Moreover, these automated routines assume T_1_‐induced signal recovery to equilibrium and steady‐state signal behavior, and are therefore, ill‐suited for use with non‐equilibrium magnetization inherent to HP MR. Additionally, quality assurance relies on thermally polarized xenon phantoms under pressure,^22^ but they lack insights into dissolved‐phase xenon, including individual coil loading (i.e., homogeneity) or the ^129^Xe signal's precise frequency in the heterogeneous lung environment.^15^ To account for variations in the coil performance and subject variation, it is necessary to perform scan calibrations on an individual basis.

The XeCTC position manuscript^15^ recommends a dedicated scan that provides the data needed to calculate nominal/global flip angle setting, center frequency of the gas phase, and TR for dissolved phase. This scan comprises 500 acquisitions of the free induction decay (FID) with RF excitation centered at the dissolved ^129^Xe frequency (218 ppm), followed by 20 acquisitions at the gaseous frequency (0 ppm). This allows frequencies of the gas and dissolved phase resonances to be determined, as well as the timing parameters (e.g., TE, TR, and the optimal flip angle for each subject). TR strongly influences the relative ratio of red blood cell (RBC) to membrane signal and has been set to 15 ms, per the XeCTC recommendation.^15^

**FIGURE 3:**
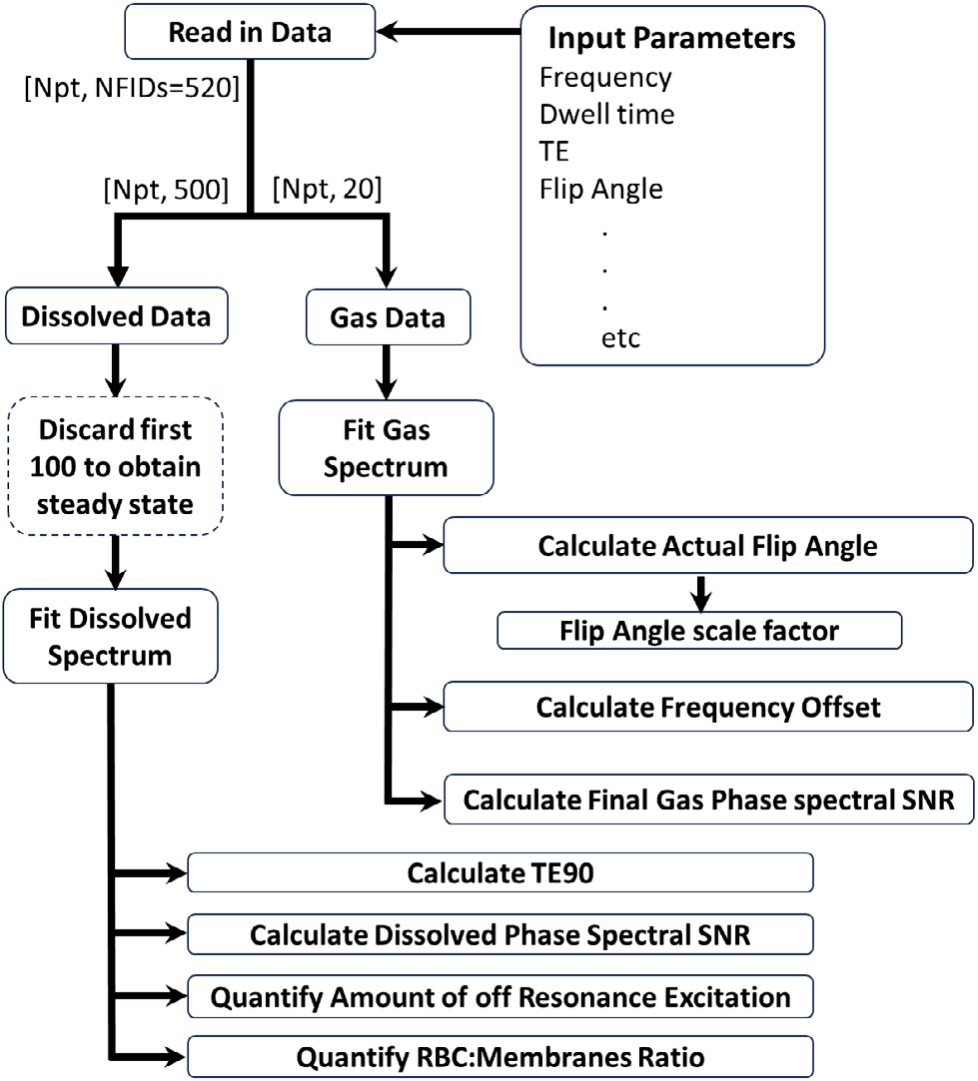
Calibration analysis pipeline for raw data acquired with the ^129^Xe MRI Clinical Trials Consortium calibration sequence.^15^ The diagram illustrates the calibration analysis process, involving gas data fitted for the flip angle scale factor and frequency offset and dissolved data fitted for the TE90 and RBC: Membrane ratio. These procedures ensure accurate measurements and reliable results for subsequent ^129^Xe scans.

Steps for the proposed calibration analysis are shown in Figure 3. The gas spectra obtained are used to calibrate the flip angle, α, by fitting the peak intensities to a simple model of gas signal decay. Assuming $T_{1}$ relaxation is negligible during data acquisition, signal decay is given by

(1) $$S_{i}=S_{0}\cos{\left(\alpha \right)}^{i-1}+C,$$

where ($S_{i}$) is magnitude of the signal intensity resulting from the *ith* RF excitation, $S_{0}$ is the signal magnitude expected from the first excitation, and C a mean of the noise offset present in magnitude data.

Once the actual flip angle delivered during the calibration sequence is determined, the prescribed flip angle can be scaled accordingly. That is, if flip angle initially prescribed to acquire calibration data is $\alpha_{p}$, but signal decay indicates an applied flip angle of $\alpha_{a}$, subsequent desired flip angles (or reference voltages) for future scans can be achieved by scaling the angle prescribed for data acquisition by $\alpha_{p}/\alpha_{a}$. The optimal flip angle for each imaging sequence is determined using equations provided in Table 1.

**TABLE 1:** Mathematical expressions for calculating the optimal flip angles for each imaging type with references provided for additional information.

Imaging type	Mathematical expression
Ventilation	Cartesian: $\alpha =\max\;\left[\sin\left(\alpha \right)\;\left(\frac{\cos{\left(\alpha \right)}^{N}-1}{\ln\cos\left(\alpha \right)}\right)\right]$ Center Out(spiral,radial): $\alpha =\max\;\left[\;\frac{\;\sin\left(\alpha \right)}{N}\left[\frac{1-\cos{\left(\alpha \right)}^{N}}{1-\cos\left(\alpha \right)}\right]\right]$(20)
Diffusion	Cartesian: $\alpha =\max\;\left[\sin\left(\alpha \right)\;\left(\frac{\cos{\left(\alpha \right)}^{N\times \mathrm{Nb}}-1}{\ln\cos\left(\alpha \right)}\right)\right]$ Center Out(spiral,radial): $\alpha =\max\;\left[\;\frac{\;\sin\left(\alpha \right)}{N}\left[\frac{1-\cos{\left(\alpha \right)}^{N\times \mathrm{Nb}}}{1-\cos{\left(\alpha \right)}^{\mathrm{Nb}}}\right]\right]$ (9)
Gas exchange	Radial: $\alpha_{\mathrm{gas}}=0.5$ (15) $\alpha_{\text{dissolve}}=20$ (15)

*Note*: N is the number of excitations, and Nb is the number of b‐values. For both ventilation and diffusion, a 2D slice‐selective approach is assumed. Additionally, the same mathematical expressions for calculating optimal flip angles can be applied to Cartesian acquisition with either linear or centric encoding order for both ventilation and diffusion.

The dissolved FIDs are used to determine the optimal TE for 1‐point Dixon gas‐exchange measurements. The initial 100 dissolved FIDs are discarded to remove the confounding effect of ^129^Xe signal in the larger vasculature downstream of the pulmonary capillary bed during inhalation.^15^ Additionally, the dissolved spectrum is fit to determine the amplitudes of the RBC and membrane peaks and the phase separation that evolved between the two peaks during the RF pulse and subsequent acquisition delay. The TE required for the RBC and membrane peaks to achieve a 90° phase separation at the k‐zero point, TE90, (assuming a center‐out trajectory such as radial acquisition) is calculated as follows:

(2) $$\mathrm{TE}90=\mathrm{TE}+\frac{90-\left(\text{Phase}\left(\mathrm{RBC}\right)-\text{Phase}\left(\text{Membrane}\right)\right)}{360\times \left(\text{Frequency}\left(\mathrm{RBC}\right)-\text{Frequency}\left(\text{Membrane}\right)\right)}.$$

where TE is the echo time used in the calibration scan.

XIPline currently supports calibration analysis using various raw data formats, including .data/.list for Philips scanners, .dat for Siemens scanners and .7 for GE scanners (Table 2). XIPline also supports International Society for Magnetic Resonance in Medicine raw data (ISMRMRD) format.^23^ New raw data formats can also be incorporated by users into the analysis pipeline. Step‐by‐step instructions for performing the calibration analysis can be found in the user's manual document in supporting documents (it can also be found here: https://github.com/aboodbdaiwi/XIPline/tree/main/user_manual).

**TABLE 2:** Currently supported data formats (XIPline V1.1.0).

Imaging type	Supported data type				
	General image formats	Raw data			
		Philips	Siemens	GE	ISMRMRD
Calibration	–	.data/.list	.dat	−.7	.h5 or .mrd
Ventilation	.dcm (single or multiple files) .mat (single): one 3D variable size (x,y,slices) .nii or .gz (single): one 3D variable size (x,y,slices)	.data/.list (2D Cartesian only)	.dat (2D Cartesian only)	−.7 (2D Cartesian only)	.h5 or .mrd (2D Cartesian only)
Diffusion	.dcm (single or multiple files): have to specify the number of b‐values and acquisition order .mat (single): one 4D variable (x,y,slices,b‐values) .nii or .gz (single): one 4D variable size (x,y,slices,b‐values)	.data/.list (2D Cartesian only)	.dat (2D Cartesian only)	−.7 (2D Cartesian only)	.h5 or .mrd (2D Cartesian only)
Gas Exchange	.mat (single): not recommended; see user's manual	.data/.list/.sin (3D radial only)	.dat (3D radial only)	.7 (3D radial only)	.h5 or .mrd (3D radial only)
Anatomical	.dcm (single or multiple files) .mat (single): one 3D variable size (x,y,slices) .nii or .gz (single): one 3D variable size (x,y,slices) See user's manual for gas exchange data	.data/.list .data/.lab/.sin (2D Cartesian or 3D radial only)	.dat (2D Cartesian or 3D radial only)	.7 (2D Cartesian or 3D radial only)	.mrd or .h5 (2D Cartesian or 3D radial only)

*Note*: ISMRMRD: International Society for Magnetic Resonance in Medicine Raw Data.

### 2.3 Image analysis

**FIGURE 4:**
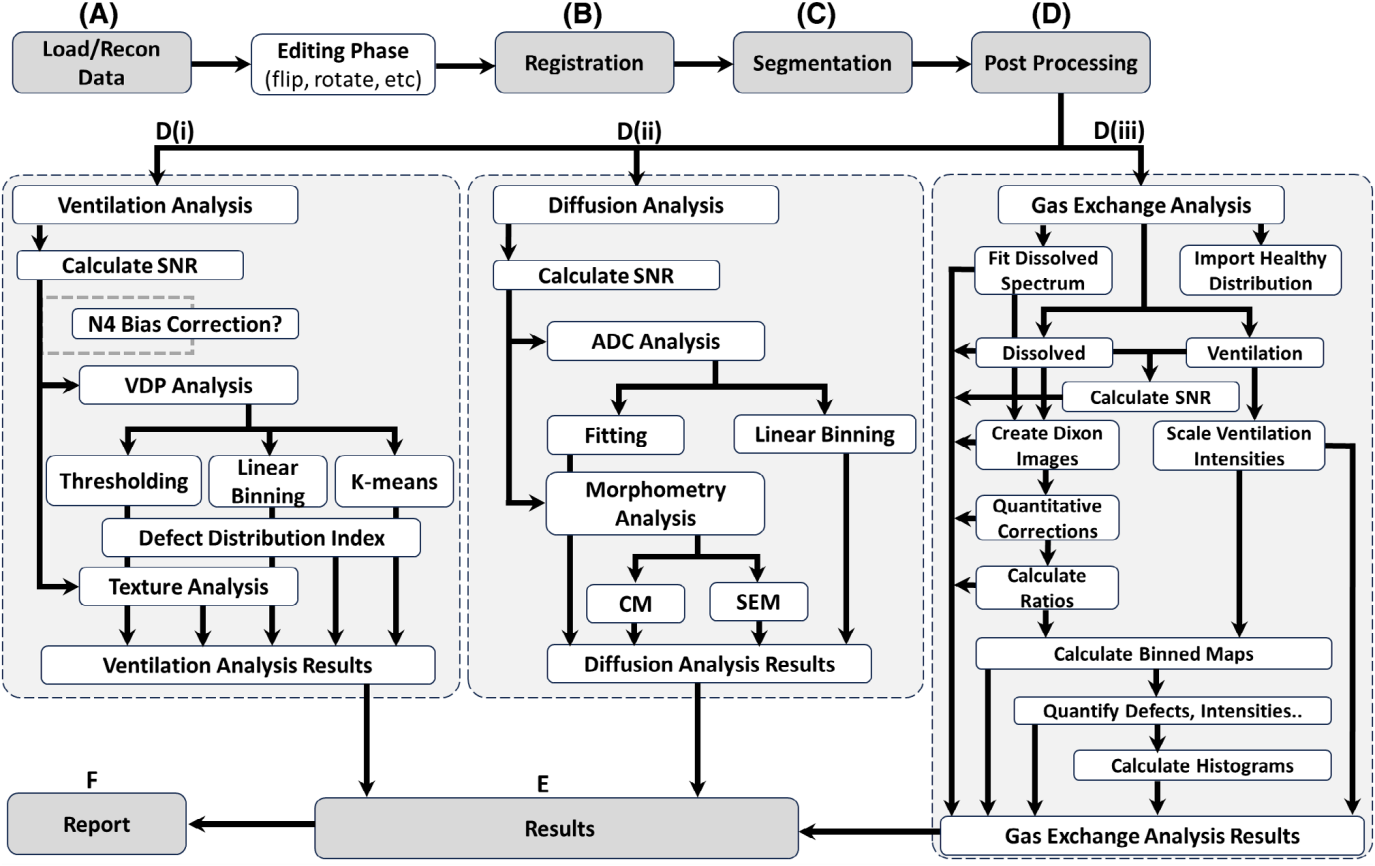
Analysis pipeline for ventilation, diffusion, and gas exchange images. The process begins by loading reconstructed images or reconstructing images from raw data (A), followed by any necessary image formatting before registering (B) the ^129^Xe and anatomical images (if available). Subsequently, the segmentation process (C) creates lung and airways masks. The post‐processing analysis is the final step (D). Ventilation analysis: (D[i]), ventilation defect percentage (VDP) analysis is conducted using thresholding, K‐means or linear binning methods. Moreover, defect distribution index (DDI) can also be calculated in 2D or 3D for a specific defect map. Texture analysis can also be performed for ventilation images. Diffusion analysis: (D[ii]), the ADC maps are generated using different fitting methods. Linear binning analysis can also be performed based on a patient's age. For multiple b‐value diffusion acquisition, morphometric analysis can be conducted using the cylindrical model (CM) or the stretched exponential model (SEM). Gas exchange analysis: (D[iii]) involves performing 1‐point Dixon analysis. All results are saved and exported (E). Optionally, a patient report can be generated as well (F).

The steps for image analysis of all HP ^129^Xe scans are shown in Figure 4. The image analysis process encompasses ventilation, diffusion‐weighted, and gas exchange. It starts with loading reconstructed or raw k‐space data. This is followed by image editing and anatomical registration to the ^129^Xe image. Next, segmentation of lungs and airways masks is performed. This leads to post‐processing analysis, which includes ventilation defect percentage (VDP), defect distribution index (DDI), and texture analysis for ventilation. Additionally, ADC and morphometry fitting for diffusion‐weighted imaging and 1‐point Dixon analysis for gas exchange are conducted. The upcoming sections provide detailed descriptions of each individual step.

#### 2.3.1 Input data

Figure S1 displays the interface and options of the “Loading Data” tab. XIPline is designed to provide compatibility with various imaging formats, including DICOM, MATLAB, NIFTI files, and more. This range of supported formats ensures easy integration with online or offline image reconstruction software. Moreover, XIPline offers support for offline reconstruction of 2D Cartesian gradient echo (2D GRE) acquisitions using .data/.list (Philips), .dat (Siemens) and .7 (GE) formats for ventilation, anatomical and diffusion imaging. XIPline also supports non‐Cartesian image reconstruction for 3D radial gas exchange data (gas and anatomical) acquired on Philips, Siemens or GE scanners.

However, the intricacy arising from diverse input raw data, including variations in format, ordering, and preprocessing, poses challenges for developing a universally applicable pipeline. Fortunately, widely used open‐source data formats, like the MR raw data (MRD)^23^ for image data, have been developed standardize data structures and facilitate efficient algorithm sharing. XIPline supports offline reconstruction of 2D GRE (for ventilation and diffusion) and 3D radial (for gas exchange) acquisitions with ISMRMRD format. These types of data format are supported for both ^129^Xe gas and anatomical ^1^H images. For a comprehensive breakdown of the supported imaging and raw data formats, please refer to Table 2. Additionally, a denoising option for xenon images is also available based on the block matching 3D method.^24^

#### 2.3.2 Registration

For ventilation and gas‐exchange imaging, it is common to acquire an anatomical proton scan with imaging parameters (e.g., patient location, voxel size, FOV, and lung inflation level^15^) that match those used to acquired xenon images. This alignment provides thoracic cavity image spatially congruent with the functional images to serve as reference for generating lung masks. Note, proton images are used exclusively for registration and as a reference for lung parenchyma segmentation, but if the “no proton images” option is selected, segmentation will rely solely on xenon images, bypassing the registration step.

Figure S2 shows the interface and options of the “Registration” tab. To facilitate image co‐registration, the multimodal 3D image registration capabilities integrated into MATLAB is used. Manual edits of registration for finer adjustments are also available. This registration technique enables anatomical images to be aligned with the corresponding HP gas image, taking advantage of the shared structural features present in both datasets. By leveraging this tool, users can achieve accurate spatial alignment between the anatomical and HP gas images, allowing for precise analysis and interpretation of the gas distribution patterns within the lungs.

#### 2.3.3 Segmentation

Figure S3 displays the interface and options of the “Segmentation” tab. XIPline offers three lung and airway segmentation approaches: manual, thresholding, and pretrained convolutional neural network (CNN) models (see Supporting Information **S1** for details of the CNN models). It is recommended to start with the deep learning‐based segmentation (pretrained models) for initial processing, followed by manual adjustments as necessary.

##### Manual Segmentation

XIPline leverages the capabilities of using free‐hand method or the volume segmenter application (a standard MATLAB app within the Image Processing Toolbox) that provides a range of powerful methods to segment the lung parenchyma and airways, including freehand drawing, painting, and thresholding. Lung segmentation can be performed in just a few simple steps. To access all features provided by this method, XIPline must be run in developer mode. However, if the complied XIPline is used, a simpler version will be employed for segmentation.

##### Threshold segmentation

XIPline also includes a convenient and simple thresholding technique based on Otsu's approach,^25^ which allows for customizable threshold level and structuring element size (default values = 1 for both, a relatively small structuring element is used, and the threshold is applied without any scaling). Additionally, this thresholding technique can be used to eliminate airways from the lung masks using a manual freehand tool included in the XIPline package.

##### Automatic segmentation

In the rapidly evolving field of HP imaging, significant advancements have been made to enhance accuracy and reproducibility, minimize human error, and reduce post‐processing time through the development of automatic lung segmentation approaches.^26^, ^27^, ^28^ XIPline provides deep‐learning models that are capable of accurately segmenting the lungs in both 2D and 3D images, considering scenarios with and without anatomical images (single or dual channel inputs^27^). To provide comprehensive information about the trained models, detailed descriptions and specifications are presented in the supplementary materials (Supporting Information **S1**).

#### 2.3.4 Post processing

##### Ventilation

Ventilation imaging using HP ^129^Xe MRI^29^ is a widely used and straightforward way to visualize regional distribution of inhaled gas in the lungs and ventilation imaging may be performed with both 2D and 3D strategies with fast gradient echo or steady state sequences.^16^, ^29^, ^30^, ^31^, ^32^, ^33^ Figure S4 shows the interface and options for the “Ventilation Analysis” tab (also see Figure 4 D[i] diagram). XIPline includes an option for N4ITK bias field correction^34^ (settings are shown in Supporting Information S2). By applying this correction, intensity variations caused by non‐uniformity in the MRI data because of coil sensitivity and regional variations in RF‐induced and T_1_‐induced signal decay are mitigated partially, therefore, enhancing the reliability and accuracy of subsequent analysis steps. SNR is calculated according to $\;S_{\text{lung}}/\sigma_{\mathrm{BG}}$, where $S_{\text{lung}}$ is mean signal amplitude within the mask; and $\sigma_{\mathrm{BG}}$ is the SD of the noise (excluding airways). $\sigma_{\mathrm{BG}}$ is calculated from the noise measured in the background of the magnitude image,$\;\sigma_{M}$, according to $\;{{\;\sigma }_{\mathrm{BG}}^{2}=\sigma }_{M}^{2}/\left(2-\pi /2\right).$ ^35^, ^36^, ^37^

Ventilation images are quantified by expressing the percentage of lung voxels with no or low signal intensity, indicating non‐ventilated or hypo‐ventilated airspaces, respectively. This measure is known as the VDP and can be defined as^38^:

(3) $$\mathrm{VDP}=\left(1-\frac{1-\mathrm{VV}}{\mathrm{TLV}}\right)\times 100\%,$$

where, VV represents the volume of the lung that is effectively ventilated, and the total lung volume (TLV) is determined through the segmentation of lung parenchyma. Several algorithms can be used to calculate VDP, including thresholding,^39^, ^40^, ^41^ linear binning,^37^, ^39^, ^42^, ^43^ hierarchical k‐means,^44^ fuzzy spatial c‐means,^45^ Gaussian mixture modeling with Markov random field,^28^ and trained CNN.^26^, ^27^, ^28^, ^46^ For the current release, thresholding, linear binning, and k‐means methods are implemented and supported in this analysis application.

Using the thresholding method,^39^, ^40^, ^41^ VDP is determined as the proportion of lung volume with signal below a specific threshold of the whole‐lung mean on ventilation images. Here, the default incomplete ventilation threshold is set to 60% of the mean whole‐lung signal, which was determined empirically to provide the maximum difference between healthy control and disease groups.^40^, ^41^ Furthermore, to enhance the accuracy of VDP quantification, a median filter, with a 3 × 3 kernel, is applied to the defect mask array. This step effectively eliminates “hot” pixels and small vessels and makes the analysis more robust to variations in SNR, which has the potential to impact the ventilation defect assessment.

In the linear‐binning method,^37^, ^39^, ^42^, ^43^ the signal from the airways is used to normalize and rescale the signal histogram, ranging from 0 to 1, using the 99th percentile of the cumulative distribution in the whole image (excluding airways). Mean (μ) and SD (σ) of the averaged rescaled intensity histogram from healthy controls were used to define the threshold values for six bin‐map boundaries (μ ± σ). These bins were designed to identify regions with ventilation defects (<μ‐2σ), low ventilation (μ‐2σ – μ‐σ), two normal ventilation bins (μ‐σ – μ + σ), and two hyper‐ventilation bins (>μ + σ). For images with no bias‐field correction, the mean and SD were calculated to be 0.54 ± 0.15, and for the same images with N4ITK bias‐field correction,^34^ the values were determined to be 0.68 ± 0.14 and set as the default values. The default values for mean and SD are for 2D images, but they can be manually adjusted for different image acquisition (e.g., 3D acquisition).

For the k‐means method, the ^129^Xe signal is categorized visually distinct classes, which typically include signal void, hypointense (or partial volume), normal intensity, and hyperintense signal. To achieve this classification, a hierarchical K‐means clustering approach was used,^44^ dividing the histogram of signal intensity frequencies into four clusters. However, the initial K‐means clustering resulted in a single cluster, C1, encompassing both signal void and hypointense regions (comprising partial volumes and/or partially ventilated volumes). To address this, K‐means clustering was reapplied in a three‐step process: (1) initial application of K‐means with four clusters to the ^129^Xe image; (2) subsequent application of K‐means with four clusters to C1; and (3) merging the first two clusters from step 2 to represent the background and ventilation defects and combining the last two clusters from step 2 to represent the hypointense signal regions.

XIPline also incorporates the DDI^47^, ^48^ to assess the 2D spatial arrangement of ventilation defects. DDI evaluates the spatial “clustering” of defect voxels in the lung, producing a defect distribution index for the entire lung. This index indicates whether defects are dispersed (low DDI) or concentrated (high DDI). This approach enables an observer‐independent evaluation of defect distribution, supporting longitudinal analysis and interindividual comparisons. Additionally, we extend the application of the DDI technique to quantify the 3D spatial distribution of ventilation defects observed in ^129^Xe imaging.

Moreover, texture analysis using the gray level run lengths method (GLRLM)^49^ metwas integrated into XIPline, enhancing its capability to quantitatively assess ventilation heterogeneity, spatial distribution, and gray‐level run patterns within the ventilation data. As part of this process, the images are normalized to a signal intensity range of 0 to 1, ensuring consistency in the quantitative measurements and allowing for a more accurate evaluation of ventilation characteristics.

##### Diffusion

Diffusion‐weighted ^129^Xe MRI is a histologically validated approach to assess lung microstructure—most commonly by calculating the ADC of the Xe gas.^50^, ^51^ By imaging ^129^Xe with multiple diffusion‐weightings (b‐values), it becomes possible to quantify morphological parameters like alveolar dimensions. In general, ^129^Xe diffusion‐weighted imaging enables the detection of age and disease‐related pulmonary microstructure changes like emphysema.^51^, ^52^, ^53^, ^54^, ^55^, ^56^, ^57^

The most common approach for diffusion‐weighted imaging involves using a 2D slice selective gradient echo sequence with bipolar diffusion gradients.^52^, ^54^, ^55^, ^58^, ^59^ In cases where only ADC is of interest, it is common to apply only two diffusion weightings (b‐values) and calculate ADC as follows:

(4) $$\mathrm{ADC}=-\frac{N_{b}\sum_{i=1}^{N_{b}}b_{i}\;\ln\left(S_{i}\right)-\sum_{i=1}^{N_{b}}b_{i}\;\sum_{i=1}^{N_{b}}\ln\left(S_{i}\right)}{N_{b}\;\sum_{i=1}^{N_{b}}b_{i}^{2}-{\left(\sum_{i=1}^{N_{b}}b_{i}\;\right)}^{2}},$$

where $N_{b}\geq 2$; $\;b_{i=1}=0$ s/cm^2^.

Here, $N_{b}$ is the number of diffusion weightings (i.e., b‐values) and $S_{i}$ is the signal for the image acquired with the specific b‐value, $\;b_{i}$.

Figure S5 shows the interface and options for the “Diffusion Analysis” tab (Figure 4 D[ii] diagram). Note, SNR is computed following the procedure outlined for ventilation analysis. XIPline enables standard ADC mapping, which includes multiple fitting options such as linear, weighted‐linear, and Bayesian methods.^36^ These fitting options enable users to model the diffusion behavior and obtain ADC maps that are suitable for their specific analysis requirements.

In addition to the standard ADC mapping, the linear‐binning method was modified^37^ to account for age dependency in the healthy reference distribution of ADC maps. The default healthy reference equations for mean and SD are μ = 0.0002 × age + 0.029 and σ = 5 × 10^−5^ × age + 0.0121 cm^2^/s.^7^ This modification ensures age‐related variations in diffusion parameters are appropriately considered during the mapping process. Importantly, these equations can be modified by the user to best serve their needs.

Finally, XIPline offers morphometry‐fitting capabilities based on two distinct models of the lung microstructure: the cylindrical model^60^ and stretched‐exponential model.^61^ These models provide the ability to extract additional parameters (e.g., acinar duct radius, alveolar sleeve depth, mean linear intercept, surface to volume ratio, alveolar density, and mean diffusive length scale) and gain deeper insights into tissue microstructure and morphology.

##### Gas exchange

HP ^129^Xe has a unique capability to dissolve into tissues, including pulmonary tissues, where it displays distinct chemical shifts in the membrane (198 ppm) and RBCs (217 ppm).^62^, ^63^ This makes it well‐suited for “gas exchange imaging”, and in the common and XeCTC‐recommended 1‐point Dixon method,^64^ gas and dissolved phase signals are acquired in an interleaved fashion using 3D radial imaging with an appropriate echo time, resulting in a 90° phase separation between RBC and membrane signals at the k‐zero point.^64^

For gas‐exchange data analysis, the generalized linear binning method using a healthy‐reference distribution was implemented.^65^ Figure S6 shows the interface and options for the “Gas‐Exchange Analysis” tab (Figure 4D[iii]). The analysis process is initiated by selecting the healthy reference distribution. The default option selects healthy distributions derived from gas exchange data at Cincinnati Children's Hospital Medical Center. Users can alternatively import a healthy distribution file in (.mat) format. Additionally, these distributions can be input manually. Initial manual values are gas = 0.51 ± 0.19, dissolved = 0.0075 ± 0.00125, membrane = 0.0049 ± 0.0015, RBC = 0.0026 ± 0.001, RBC/membrane = 0.53 ± 0.18, and RBC oscillations = 8.96 ± 10.56. After fitting the accompanying dissolved spectra, the RBC/membrane ratio, $T_{2}^{*}$ values, frequencies (gas, RBC, and membrane), and contamination factors are computed. $T_{2}^{*}$ values are determined as $\frac{1}{\pi .\text{FWHM}}*1000$ (in ms), where full width at half maximum (FWHM) of the fitted spectra for gas, membrane, and RBC.

The ^129^Xe dissolved‐phase image is separated into distinct RBC and membrane images, using the Dixon method.^64^, ^66^ First, a phase map derived from the single‐resonance ^129^Xe gas‐phase image is used to account for any phase shift due to B_0_ inhomogeneities. In this process, the dissolved ^129^Xe image, captured at TE90, is phase‐shifted to align the RBC and membrane signals within the real and imaginary components of the dissolved image. RBC has zero phase, and membrane has 90° phase, effectively confining the signal at k‐zero to the real and imaginary portions of the data, respectively. The specific phase shift was chosen such that the overall RBC:membrane ratio, derived from the B_0_‐corrected image, corresponded to the ratio obtained from whole‐lung spectroscopy. Because the RBC, membrane, and gas signal intensities are affected by $T_{2}^{*}$ to different extents, the image intensities are adjusted to account for the relaxation before the imaging TE. Following these corrections, any residual negative signal of negligible magnitude was set to zero.

For quantitative maps, the raw intensities in RBC, membrane, and gas images are corrected to account for $T_{2}^{*}$ decay caused by signal loss before the TE90 according to $I=I/e^{\left(-\mathrm{TE}90/T_{2}^{*}\right)}$. Furthermore, the intensity of the ^129^Xe gas‐phase image are scaled by a factor of $I=I*\frac{\sin\left(\alpha_{\text{diss}}\right)}{\sin\left(\alpha_{\mathrm{gas}}\right)}=\frac{\sin\left(20^{{}^{\circ}}\right)}{\sin\left(0.5^{{}^{\circ}}\right)}=39$, compensating for the higher flip angle applied to the dissolved images compared to the gas‐phase images. Last, the $T_{2}^{*}$‐corrected RBC and membrane images are divided by the $T_{2}^{*}$‐ and flip‐angle‐adjusted ^129^Xe gas image. This yields RBC:gas and membrane:gas maps to normalize polarization discrepancies and variations in inhaled gas volume across subjects. Additionally, the high‐resolution gas‐phase image itself is normalized, anchored by its 99th percentile of intensities similar to that done in the above dedicated ventilation analysis.^43^, ^66^

All images are subsequently categorized into bins based on the mean and SD of the healthy distributions. Gas, dissolved, and RBC images are distributed into six bins, whereas the membrane images are distributed into eight bins because of the elongated distribution tail.^66^ SNR is calculated according to $\;\left(S_{\text{lung}}-S_{\mathrm{BG}}\right)/\sigma_{\mathrm{BG}}$, where $S_{\text{lung}}$ is mean signal amplitude within the mask; $S_{\mathrm{BG}}$ is the mean of the noise; and $\sigma_{\mathrm{BG}}$ is the SD of the noise (excluding airways).

It is important to note that the gas‐exchange analysis in the initial XIPline release (V1.1.0) is customized to suit the specific requirements of our center's data. However, the gas‐exchange analysis was implemented based on the XeCTC guidelines. Users also have the freedom to edit the analysis code and customize it according to the specific characteristics of their gas‐exchange data.

### 2.4 Open research statement

XIPline provides both compiled, which does not require MATLAB installation or license, and source code access (available at https://github.com/aboodbdaiwi/XIPline), offering users a ready‐to‐use application or the freedom to customize it. This emphasis on open‐source principles not only promotes repeatability, version tracking, and full method transparency, but also paves the way for seamless multi‐site studies with harmonized data analysis. As a result, XIPline can become a catalyst for fostering collaboration, moving toward a consensus analysis, and offering a range of diverse user experiences.

Note, a comprehensive tutorial (videos) on installing and using the XIPline application is available at the following link: https://www.youtube.com/@user‐bd4qv6eg4i.

### 2.5 Ethics statement and imaging protocol

The images presented in the results were obtained from data collected under protocols approved by the institutional review board at Cincinnati Children's Hospital (IND 123577). Written informed consent or assent was obtained from all participants or their guardians for pediatric subjects undergoing ^129^Xe MRI. Isotopically enriched xenon (85% ^129^Xe, Linde Elec and Specialty Gases) was polarized to 10% to 40%^30^ using Polarean polarizers (Polarean Imaging). The xenon dose administered was 1 L for adults or 1/6th of the estimated total lung capacity for pediatric subjects, with a maximum dose of 1 L, adhering to American Thoracic Society guidelines.^31^ ^129^Xe images were captured during a breath‐hold of up to 16 s post‐inhalation. A medical professional monitored heart rate and oxygenation throughout the procedure. Imaging was conducted using a 3 T Philips scanner (Philips Healthcare). Additionally, a ^1^H scan was performed during a separate breath‐hold with a volume‐matched air bag for thoracic cavity segmentation, using the same imaging sequence.

## 3 RESULTS

### 3.1 Calibration analysis

**FIGURE 5:**
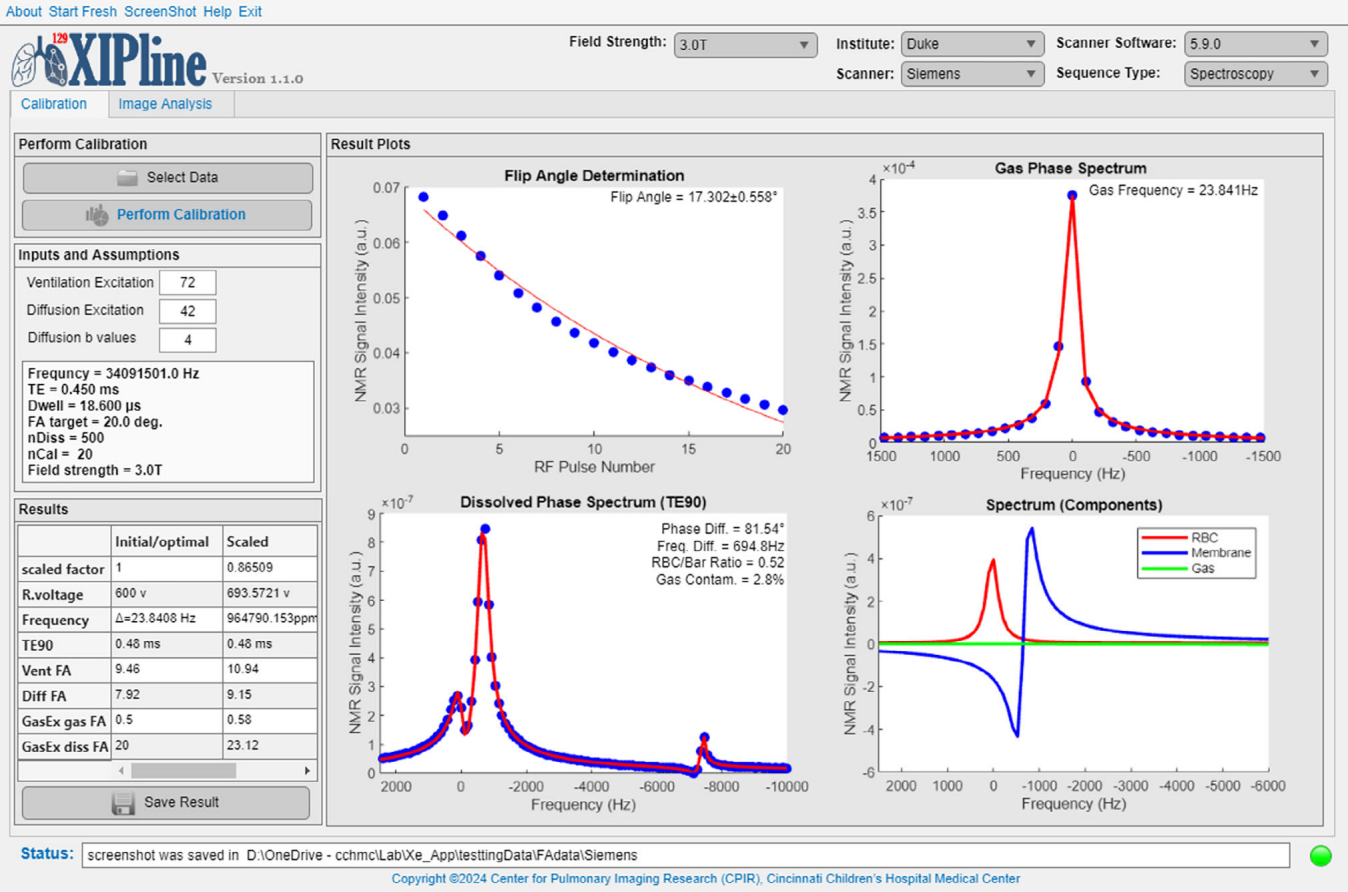
Calibration analysis results. The calibration input parameters are shown in the top‐left panel, which can be adjusted both before and after conducting the analysis. The optimal flip angles and frequency offset are depicted in the bottom‐left panel. On the right side, graphs display the fitting of gas and dissolved spectra. The top‐left graph illustrates the gas signal decay fitting, used to extract the flip angle scale factor. On the top‐right graph, the gas phase spectrum is fit to determine the frequency offset. The bottom‐left graph shows the dissolved phase spectrum, along with the red blood cells (RBC):membrane ratio. Last, the bottom‐right graph demonstrates the overall spectrum of RBC, membrane, and gas.

An example of the results from the calibration analysis on real in‐vivo data is shown in Figure 5. In the top‐left panel of the Calibration tab, the calibration input parameters (number of excitations, number of b‐values, etc) are shown, which can be adjusted both before and after conducting the analysis. In the bottom‐left panel of the Calibration tab, the scaling factor, frequency offset, and the optimal flip angles are displayed. For non‐Cartesian sequences, the optimal flip angle is calculated based on maximizing the ^129^Xe signal.^67^

On the right side, a series of informative graphs permit visual assessment of the quality of the data. The intensities of the gas FIDs (to extract the flip angle scale factor) are shown in the top‐left graph. On the top‐right graph, the gas‐phase spectrum is fit to determine the frequency offset. The bottom‐left graph shows the dissolved‐phase spectrum, providing valuable insights into the RBC:membrane ratio and the quality of the fit to the spectrum. Finally, the bottom‐right graph shows the individual components that contribute to the acquired spectrum encompassing the RBC, membrane, and gas components, and is used to calculate the TE90.

### 3.2 Ventilation analysis

**FIGURE 6:**
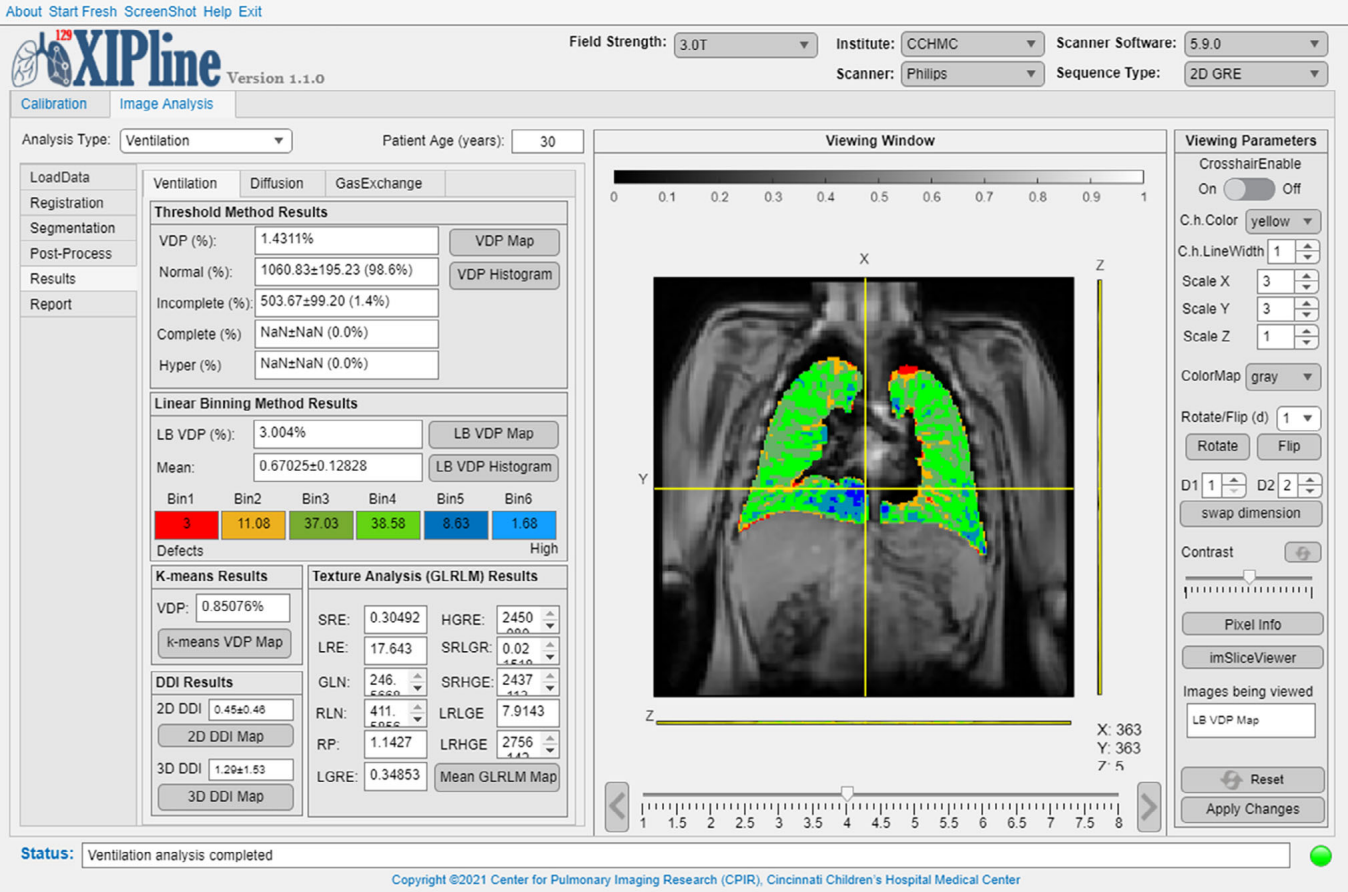
Ventilation analysis results. Summary of the ventilation analysis results are shown in the left panel of the results tab, including the mean and SD of VDP for thresholding, linear binning, and k‐means analysis methods. Additionally, VDP maps can be displayed. Moreover, mean, SD and maps of DDI for both 2D and 3D are also displayed, with the option to view the DDI maps. The mean parameters of the texture analysis are also presented, with the option to view the map of all parameters.

In Figure 6, the left panel of the Results tab provides a comprehensive summary of the ventilation analysis. Notably, VDP as well as the mean and SD of the signal intensities are shown for the chosen VDP analysis methods. In addition to numerical summaries, the visualization of VDP maps and corresponding histograms are available (Figure S8A,B), providing a visual representation of the distribution of ventilation defects and deeper characterization of the spatial patterns and severity of non‐ventilated or hypo‐ventilated airspaces. Moreover, mean, SD and maps of DDI for both 2D and 3D are also displayed, with the option to view the DDI maps.

Moreover, comprehensive analyses extend to the computation and visualization of additional metrics, encompassing the mean, SD, and maps of the DDI for both 2D and 3D approaches. Users are provided with the flexibility to explore DDI maps, enhancing their understanding of defect distribution patterns.

Furthermore, the mean texture analysis parameters are shown, offering a comprehensive overview of the lung's ventilation characteristics. For a more detailed examination, an option to view the mean of all parameters is available (Figure S8C), allowing users to explore the finer nuances of the ventilation patterns throughout the lung. The file outputs for the ventilation analysis are shown in Figure S9.

### 3.3 Diffusion analysis

**FIGURE 7:**
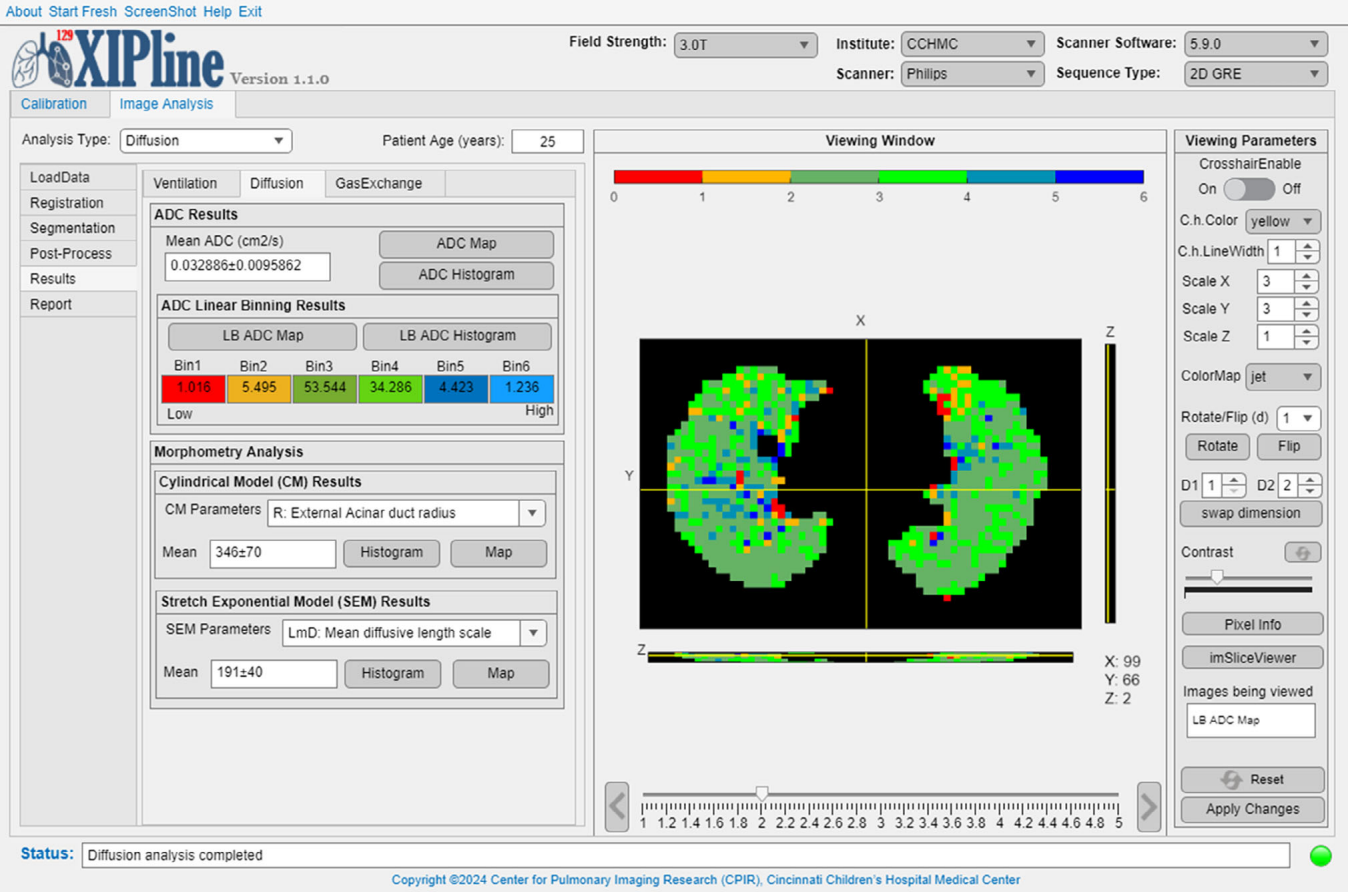
Diffusion analysis results. Summary of the diffusion analysis results are shown in the left panel of the results tab, including the mean and SD of ADC. ADC maps can also be displayed. Additionally, the results from the linear binning diffusion analysis consist of the percentage of ADC values falling into each bin, are also displayed, with an option to view the maps and the histograms. Furthermore, a summary of the morphometry analysis is available, featuring mean, SDs, maps, and histograms.

In Figure 7, the Results tab for the diffusion analysis is shown, including the whole‐lung mean and SD of the ADC maps. These key statistical measures provide a concise overview of the global gas diffusion behavior and its variability across the lungs. Moreover, ADC maps and corresponding histograms (Figure S10) provide a visual representation of the distribution of diffusion coefficients and aid in the identification of regional variations or anomalies within the patient lungs. Additionally, the outcomes of the age‐adjusted linear binning diffusion analysis include the distribution of ADC values across various bins, presented as a percentage. Users have the option to visualize both maps and histograms depicting this distribution. The Results tab also features a summary of the morphometry analysis. This includes mean and SDs for the morphometric outcomes, as well as corresponding maps and histograms, providing insights into the structural characteristics of the data. The file outputs for the diffusion analysis are shown in Figure S11.

### 3.4 Gas exchange analysis

**FIGURE 8:**
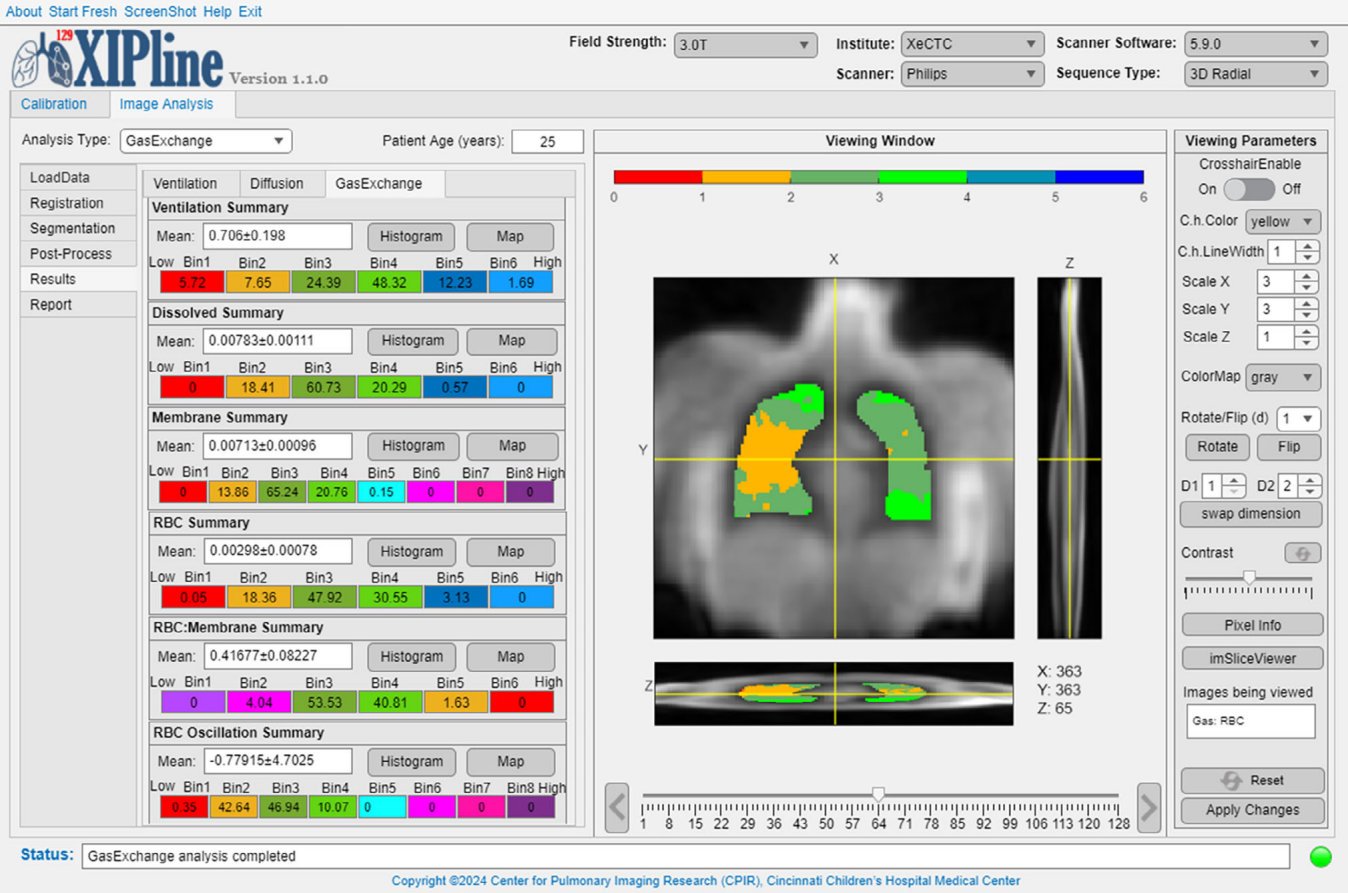
Gas exchange analysis results. In the left panel of the results tab, a comprehensive summary is presented, featuring the mean and SDs of ventilation, dissolved, membrane, red blood cells (RBC), and oscillations. The linear binning analysis method is used. Additionally, maps and corresponding histograms can be viewed, providing valuable insights into gas exchange efficiency and distribution.

In Figure 8, the Results tab for an example gas‐exchange analysis is shown. Specifically, the left panel includes essential statistical measures, such as the mean and SDs of ventilation, total dissolved phase, membrane‐uptake, RBC‐transfer, and RBC oscillations maps as well as the percentage of lung in each bin. To enrich the analysis, maps and corresponding histograms (Figure S12) provide valuable spatial representation of the distribution and variation of gas exchange components of the data being processed. The file outputs for the gas exchange analysis are shown in Figure S13. The ratio (membrane:gas, RBC:gas) maps are also calculated and exported in the final report.

## 4 DISCUSSION

XIPline offers a robust and intuitive platform specifically designed for the analysis and visualization of HP ^129^Xe MRI data. Following progress in standardizing data acquisition,^15^ this application is designed to initiate the same for analysis methods to facilitate harmonization in multi‐site studies and encourage the broader utilization of HP ^129^Xe MRI as a valuable clinical‐research tool.

Developed in MATLAB, a widely used language for MRI data analysis, XIPline allows for swift implementation of new functions and code modifications. Moreover, XIPline can be compiled as stand‐alone installers, catering to users without access to MATLAB and ensuring wider accessibility.

The ongoing development of XIPline aims to streamline the implementation of novel techniques across research sites. By providing an open‐source framework via GitHub, developers can collaborate and share their analysis techniques, reducing redundant efforts and optimizing the analysis workflow across multiple sites. This collaborative approach not only benefits experienced researchers, but also accelerates the training process for newcomers, facilitating efficient and standardized analyses.

### 4.1 Limitations and future work

The current XIPline release offers limited compatibility with raw data from various vendors, and future work will focus on accommodating data from a wider range of vendors to enhance the application's functionality. Additionally, users have the flexibility to incorporate their own image‐reconstruction algorithms. The current XIPline also lacks the support for non‐Cartesian image reconstruction, except for 3D radial gas‐exchange data. To address this, future work will focus on incorporating tools for non‐Cartesian imaging, particularly for spiral acquisition, which has many benefits including rapid acquisition, high resolution, and B_1_ field corrections.^6^, ^20^, ^68^, ^69^ However, XIPline supports 2D Cartesian and 3D radial ISMRMRD formats. This would offer a significant advantage, as users could convert their raw data into the standardized ISMRMRD format for processing by XIPline.

Although this application demonstrates the effectiveness of the implemented methods for global analysis of ventilation, diffusion, and gas exchange, it is important to acknowledge that regional analysis at the lobar or segmental level was not included. This represents a significant limitation, as region‐specific analysis, such as lobar or segmental VDP and gas exchange, could provide more detailed insights into the heterogeneity of lung function. These advanced analyses are particularly relevant for clinical applications such as image‐guided bronchial thermoplasty and bronchoscopy, where precise localization of functional impairments is critical. Future work will focus on developing and integrating methods for regional analysis within the existing framework. This will include refining the software to allow for automated segmentation and analysis of specific lung regions, thereby enhancing the precision and clinical applicability of the findings.

Another limitation of the current version of XIPline is that it does not support switching between coronal, axial, and sagittal planes for 2D images; the images are displayed based on their original acquisition plane. Although it is possible to consider interpolation methods to account for slice thickness and improve visualization, this could lead to inaccuracies because of the limitations associated with interpolating between slices of varying resolutions. Future developments could explore more advanced methods to address this issue and enhance the accuracy and flexibility of image registration and visualization within the application.

Additionally, numerous enhancements and analyses are forthcoming. Examples of these include the examination of cardiogenic oscillations derived from spectroscopy data, exploration of pulmonary capillary dynamics,^5^ and introduction of tailored analysis reports designed for easy interpretation by clinicians.

## 5 CONCLUSIONS

XIPline provides a comprehensive and user‐friendly platform for analyzing and visualizing HP ^129^Xe MRI data. Standardizing data acquisition and analysis methods will enable multi‐site clinical trials with harmonized analysis across vendor platforms and simplify the adoption of HP ^129^Xe MRI as a valuable endpoint in research and clinical settings. The modular and open‐source nature of XIPline ensures its adaptability and encourages the integration of new analysis techniques as the field progresses.

## CONFLICT OF INTEREST STATEMENT

M.M.W., J.C.W., P.J.N., and L.W.W. are consultants to Polarean.

## Supporting information


**Figure S1.** Interface and options of the Loading Data Panel. The Xenon data's location and name are determined by using the “Select Gas Data” button. Depending on the file extension of the chosen file (.dcm, .nii, etc.), the suitable function is automatically selected to load the data. A denoising option for xenon images is also available based on the Block Matching 3D (BM3D) method. If anatomical images are not available, the “No Proton” option should be chosen. Otherwise, the anatomical data location and name are specified using the “Select Anatomical Data” button. Subsequently, all data will be imported through the “Load/Read Data” button located at the bottom. The preferred images can then be visualized using the “View Images” panel. For additional details, please refer to the user's manual document.
**Figure S2.** Interface and options of the Registration Panel. If anatomical images are available, registering the proton to xenon images becomes feasible. Users can choose the transformation type and specify image resolution for precise registration. Opting for the “Use Ratio” feature allows setting image resolution as the ratio of the proton image size over the xenon image size. In cases where the number of slices between xenon and proton images differs, users can select start and end slice indices based on lung anatomy. Linear interpolation is then executed to match the number of slices before registration. Finally, registration can be initiated with the “Perform Registration” button. The registered images can then be visualized using the “View Images” panel. For additional details, please refer to the user's manual document.
**Figure S3.** Interface and options of the Segmentation Panel. Segmentation of the lung and airways mask is done through various methods, including manual, thresholding, and automatic (deep learning‐based) approaches. Additionally, pre‐existing masks can be loaded and modified as needed. For additional details, please refer to the user's manual document.
**Figure S4.** Interface and options of the Ventilation Post Process Panel. Ventilation defect percentage (VDP) and texture analysis can be performed on ventilation images. Users have the flexibility to adjust specific threshold values for the threshold method. In the linear binning method, mean and standard deviation parameters from a healthy reference can be modified. Defect distribution index can also be performed in 2D or 3D for a specific defect map. Texture analysis, utilizing the Gray Level Run Lengths Method (GLRLM) (49), is also integrated into the process. (GLRLM). For additional details, please refer to the user's manual document.
**Figure S5.** Interface and options of the Diffusion Post Process Panel. The diffusion analysis encompasses the generation of the apparent diffusion coefficient (ADC) and morphometry parameter maps (e.g., mean linear intercept, surface‐to‐volume ratio, and alveolar density, etc). ADC maps can be generated through log‐linear fitting, weighted‐linear fitting, non‐linear fitting, or Bayesian methods. Morphometry analysis relies on cylindrical and stretch‐exponential models. Additionally, linear binning analysis can be conducted using age‐adjusted mean and standard deviation values from a healthy reference (equations can be modified by the user). For additional details, please refer to the user's manual document.
**Figure S6.** Interface and options of the Gas Exchange Post Process Panel. Three options in the dropdown menu for selecting the healthy reference distributions: default, import, and manual. Default will select the healthy distributions based on gas exchange data from Cincinnati's Children's Hospital Center. The user can also import a healthy distribution file in (.mat) format; otherwise, manual values are provided, and users have the option to modify these values as needed. For additional details, please refer to the user's manual document.
**Figure S7.** Interface and options of the Patient Report Panel. A PDF report will be automatically generated, incorporating information supplied by the data analyst (as illustrated in the figure) and the results of the image analysis.
**Figure S8.** (A) An example of a VDP histogram resulted from the VDP threshold method. (B) An example of a VDP histogram resulted from the VDP linear binning method. (C) An example of mean parameters of gray level run lengths method (GLRLM) (49) analysis.
**Figure S9:** Ventilation analysis file output. The main analysis results will be saved in a folder named “Ventilation Analysis.” Within this folder, two subfolders will be present, one for the VDP analysis and another for the texture analysis. Additionally, the main analysis output will be saved as a .mat file, while a report summarizing the findings will be saved as a .pptx file. This comprehensive file organization ensures easy access to the results and facilitates further examination and documentation of the ventilation analysis outcomes.
**Figure S10.** (A) An example of an ADC histogram resulted from log‐linear fitting method. (B) An example of an ADC histogram resulted from linear binning method.
**Figure S11:** Diffusion analysis file output. The main analysis results will be saved in a folder named “Diffusion Analysis.” The main analysis output will be saved as a .mat file, while a report summarizing the findings will be saved as a .pptx file.
**Figure S12.** (A) An example of gas‐ventilation histogram resulted from linear binning method. (B) An example of membrane:Gas histogram resulted from linear binning method. (C) An example of RBC:Gas histogram resulted from linear binning method. (D) An example of RBC:Membrane histogram resulted from linear binning method. (E) An example of RBC Oscillation histogram resulted from linear binning method.
**Figure S13:** Gas Exchange analysis file output. The main analysis results will be saved in a folder named “Gas Exchange Analysis.” The main analysis output will be saved as a .mat file, while a report summarizing the findings will be saved as a .pptx file.

## Data Availability

The compiled application, along with the open‐source code and sample data, can be accessed here: https://github.com/aboodbdaiwi/XIPline. We invite users to engage with us by asking questions, reporting issues, and collaborating on this repository. Additionally, a comprehensive tutorial, including video guides on installing and using the XIPline application, is available at the following link: https://www.youtube.com/@user‐bd4qv6eg4i.
