# Supplementary material for: 129Xe Image Processing Pipeline: An open‐source, graphical user interface application for the analysis of hyperpolarized 129Xe MRI
Source: Magn Reson Med. 2024 Oct 31;93(3):1220–37. doi: 10.1002/mrm.30347 (PMC11680735; doi:10.1002/mrm.30347)
Supplement: Supplementary file 1 — Figure S1. Interface and options of the Loading Data Panel. The Xenon data's location and name are determined by using the “Select Gas Data” button. Depending on the file extension of the chosen file (.dcm, .nii, etc.), the suitable function is automatically selected to load the data. A denoising option for xenon images is also available based on the Block Matching 3D (BM3D) method. If anatomical images are not available, the “No Proton” option should be chosen. Otherwise, the anatomical data location and name are specified using the “Select Anatomical Data” button. Subsequently, all data will be imported through the “Load/Read Data” button located at the bottom. The preferred images can then be visualized using the “View Images” panel. For additional details, please refer to the user's manual document. Figure S2. Interface and options of the Registration Panel. If anatomical images are available, registering the proton to xenon images becomes feasible. Users can choose the transformation type and specify image resolution for precise registration. Opting for the “Use Ratio” feature allows setting image resolution as the ratio of the proton image size over the xenon image size. In cases where the number of slices between xenon and proton images differs, users can select start and end slice indices based on lung anatomy. Linear interpolation is then executed to match the number of slices before registration. Finally, registration can be initiated with the “Perform Registration” button. The registered images can then be visualized using the “View Images” panel. For additional details, please refer to the user's manual document. Figure S3. Interface and options of the Segmentation Panel. Segmentation of the lung and airways mask is done through various methods, including manual, thresholding, and automatic (deep learning‐based) approaches. Additionally, pre‐existing masks can be loaded and modified as needed. For additional details, please refer to the user's manual doc [file MRM-93-1220-s001.docx]

**Supporting Information**

^129^**X**e **I**mage **P**rocessing **P**ipe**line** (XIPline): An Open-Source, Graphical User Interface Application for the Analysis of Hyperpolarized ^129^Xe MRI

**Abdullah S. Bdaiwi^1^**, Matthew M. Willmering^1^, Joseph W. Plummer^1,2^, Riaz Hussain^1^, David J. Roach^1^, Juan Parra-Robles^1^, Peter J. Niedbalski^3-5^, Jason C. Woods^1,6,7^, Laura L. Walkup^1,2,6,7^, and Zackary I. Cleveland^1,2,6,7, #^

**Affiliations:**

^1^Center for Pulmonary Imaging Research, Division of Pulmonary Medicine, Cincinnati Children’s Hospital Medical Center, Cincinnati, OH 45229

^2^Department of Biomedical Engineering, University of Cincinnati, Cincinnati, OH 45221

^3^Division of Pulmonary, Critical Care, and Sleep Medicine, Department of Internal Medicine, University of Kansas Medical Center, Kansas City, KS, USA

^4^Department of Bioengineering, University of Kansas, Lawrence, KS, USA

^5^Hoglund Biomedical Imaging Center, University of Kansas Medical Center, Kansas City, KS, USA

^6^Department of Pediatrics, University of Cincinnati, Cincinnati, OH, United States

^7^Imaging Research Center, Department of Radiology, Cincinnati Children’s Hospital Medical Center, Cincinnati, OH 45229

A comprehensive tutorial (videos) on installing and using the XIPline application is available at the following link: <https://www.youtube.com/@user-bd4qv6eg4i>

**Appendix S1 | Convolutional Neural Networks Framework for Lung Segmentation**

*Hyperpolarized gas and anatomical MRI acquisition (training data):*

Hyperpolarized gas MRI acquisition involved using hyperpolarized ^129^Xe gas with 1-liter volume for adults or 1/6 of TLC for pediatrics, approximately 35% polarization, and 85% isotopic-enrichment. The gas was polarized using the Polarean 9820A Hyperpolarizer from Polarean Imaging PLC, Durham, NC. The MRI scans were performed on a Philips 3T Ingenia MRI scanner (Philips Healthcare, Best, Netherlands) using either 2D (ventilation and diffusion) or 3D (gas exchange) volumetric imaging with full lung coverage. Settings for 2D images included: FOV=200-400 mm^2^; resolution=3x3 mm^2^ (ventilation) or resolution=5x5 mm^2^ (diffusion), slice thickness=15 mm, slices=5-20 using either 2D gradient recall echo or spiral sequences. Settings for 3D images included: FOV=325^3^ mm^3^; resolution=5.8^3^ mm^3^, using radial sampling sequence.

*2D Ventilation Model (coronal and axial slice orientation):*

For the 2D model, a classic U-net model was employed on a dataset comprising 147 datasets with coronal view and 262 datasets axial view images of ^129^Xe and ^1^H anatomical images (for both single and dual channel inputs). The dataset was randomly split into 80:10:10% for training: validation: testing. The ground-truth segmentations were manually drawn by expert readers.

*2D Diffusion Model (axial slice orientation):*

For the 2D model, a classic U-net model was employed on a dataset comprising 120 datasets with axial view images of ^129^Xe. The dataset was randomly split into 80:10:10% for training: validation: testing. The ground-truth segmentations were manually drawn by expert readers.

*3D Model Gas Exchange (isotropic):*

Similarly, for the 3D model, a classic U-net model was used on a dataset of 377 subjects with isotopic images of ^129^Xe and ^1^H anatomical images (for both single and dual channel inputs). The dataset was randomly split into 80:10:10% for training: validation: testing. The ground truth segmentations were manually drawn by expert readers.

*Data Augmentation:*

To augment the data, constrained random rotation and scaling were applied, where rotation angles ranged from -10° to 10° and scaling varied from -10% to 10%. Random values were generated for each rotation axis and scaling factor during augmentation.

*Training:*

The training process utilized the Adam optimizer with a batch size of 32 for 2D and 2 for 3D, a learning rate of 0.0001, and ReLU activation. The 2D model underwent 2000 epochs during training, while the 3D model went through 1000 epochs. Training on a 16 GB NVIDIA RTX A4000 took around ~48 hours for 2D and 144 hours for 3D. Additionally, generating lung masks for new subjects using the trained model required approximately thirty seconds on a single GPU.

**Appendix S2 | N4 Bias Correction Settings**

**% X Correction**

cmd = ['"',N4Path,'N4BiasFieldCorrection.exe"',...%run bias correction

' -d 3 -i "',parentPath,'CorrectedImage.nii"',... % set to 3 dimensions and input image of Image.nii

' -s 1',... % shrink by factor of 1

' -x "',parentPath,'Mask.nii" ',... % import mask called Mask.nii

' -w "',parentPath,'Weight.nii" ',... % import mask called Weight.nii

' -c [25,0]',... % convergence

' -b [1x14x1,3]',... % spline settings %hf, lr, ap

' -t [0.75,0.01,100]',... % histogram settings

' -o ["',parentPath,'CorrectedImage.nii","',parentPath,'Bias.nii"]'];

system(cmd);

**% Y Correction**

cmd = ['"',N4Path,'N4BiasFieldCorrection.exe"',...%run bias correction

' -d 3 -i "',parentPath,'CorrectedImage.nii"',... % set to 3 dimensions and input image of Image.nii

' -s 1',... % shrink by factor of 1

' -x "',parentPath,'Mask.nii" ',... % import mask called Mask.nii

' -w "',parentPath,'Weight.nii" ',... % import mask called Weight.nii

' -c [25,0]',... % convergence

' -b [14x1x1,3]',... % spline settings %hf, lr, ap

' -t [0.75,0.01,100]',... % histogram settings

' -o ["',parentPath,'CorrectedImage.nii","',parentPath,'Bias.nii"]'];

system(cmd);

**% Z Correction**

cmd = ['"',N4Path,'N4BiasFieldCorrection.exe"',...%run bias correction

' -d 3 -i "',parentPath,'Image.nii"',... % set to 3 dimensions

' -s 1',... % shrink by factor of 1

' -x "',parentPath,'Mask.nii" ',... % import mask called Mask.nii

' -w "',parentPath,'Weight.nii" ',... % import mask called Weight.nii

' -c [25,0]',... % convergence

' -b [1x1x14,3]',... % spline settings %hf, lr, ap

' -t [0.75,0.01,100]',... % histogram settings

' -o ["',parentPath,'CorrectedImage.nii","',parentPath,'Bias.nii"]'];

system(cmd);


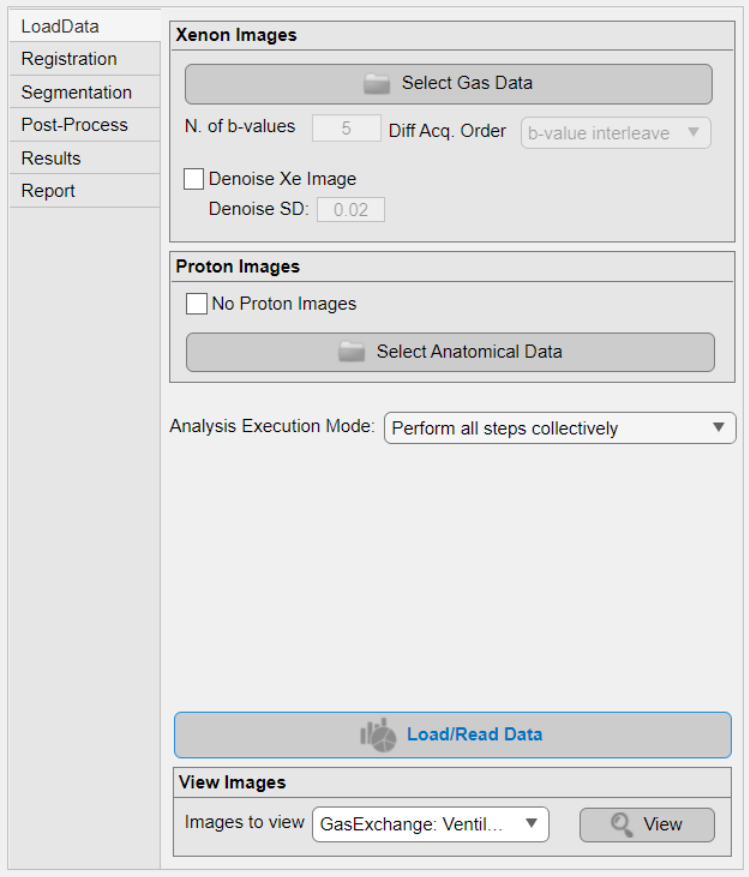


Figure S1. Interface and options of the Loading Data Panel. The Xenon data's location and name are determined by using the "Select Gas Data" button. Depending on the file extension of the chosen file (.dcm, .nii, etc.), the suitable function is automatically selected to load the data. A denoising option for xenon images is also available based on the Block Matching 3D (BM3D) method. If anatomical images are not available, the "No Proton" option should be chosen. Otherwise, the anatomical data location and name are specified using the "Select Anatomical Data" button. Subsequently, all data will be imported through the "Load/Read Data" button located at the bottom. The preferred images can then be visualized using the "View Images" panel. For additional details, please refer to the user's manual document.


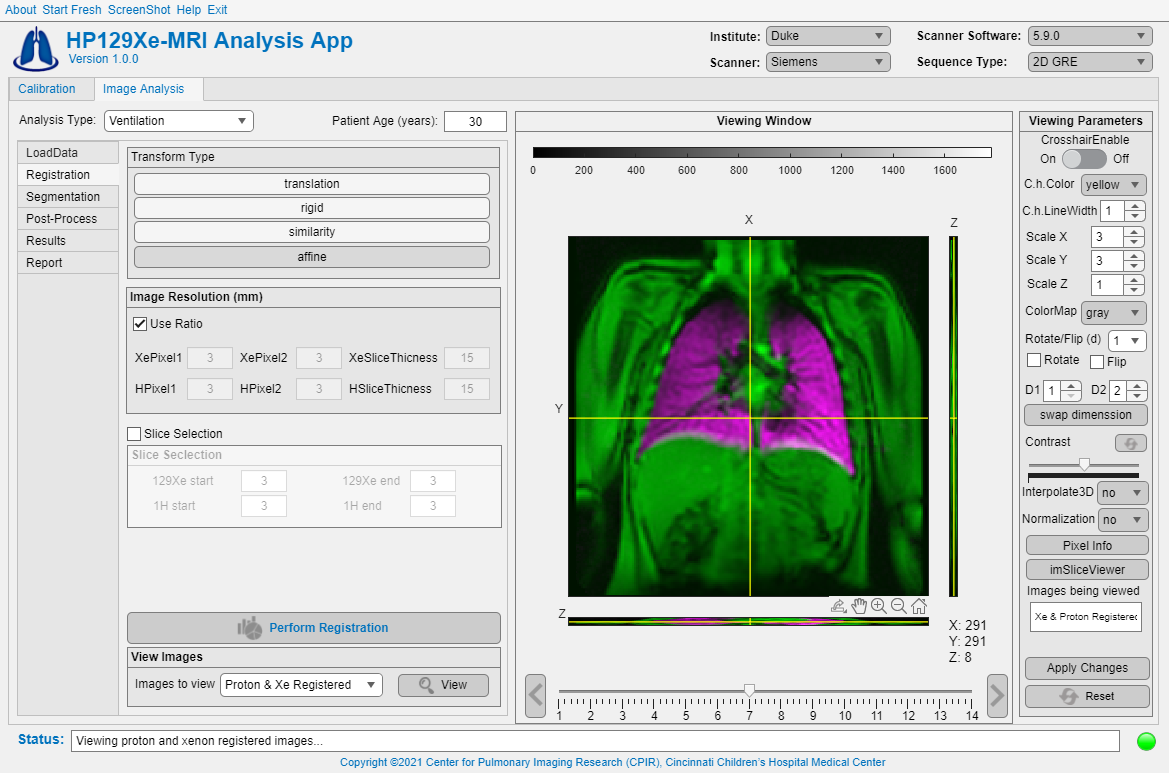
Figure S2. Interface and options of the Registration Panel. If anatomical images are available, registering the proton to xenon images becomes feasible. Users can choose the transformation type and specify image resolution for precise registration. Opting for the "Use Ratio" feature allows setting image resolution as the ratio of the proton image size over the xenon image size. In cases where the number of slices between xenon and proton images differs, users can select start and end slice indices based on lung anatomy. Linear interpolation is then executed to match the number of slices before registration. Finally, registration can be initiated with the "Perform Registration" button. The registered images can then be visualized using the "View Images" panel. For additional details, please refer to the user's manual document.


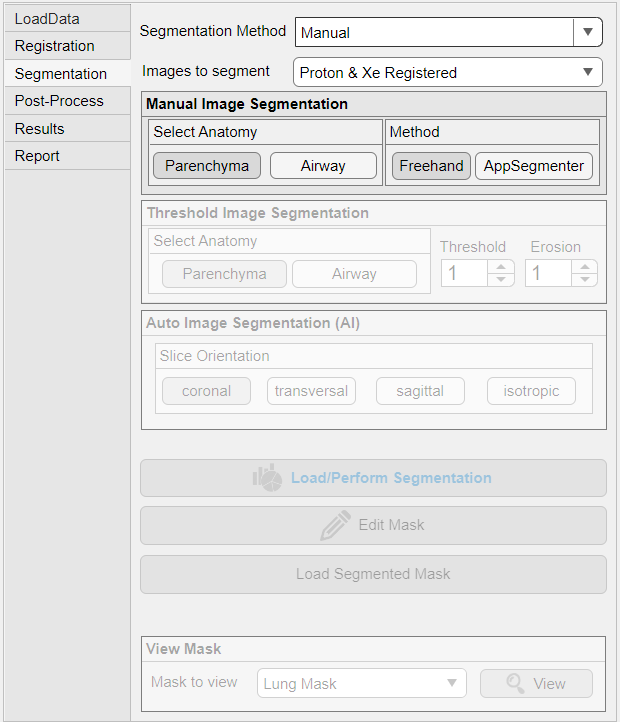
Figure S3. Interface and options of the Segmentation Panel. Segmentation of the lung and airways mask is done through various methods, including manual, thresholding, and automatic (deep learning-based) approaches. Additionally, pre-existing masks can be loaded and modified as needed. For additional details, please refer to the user's manual document.


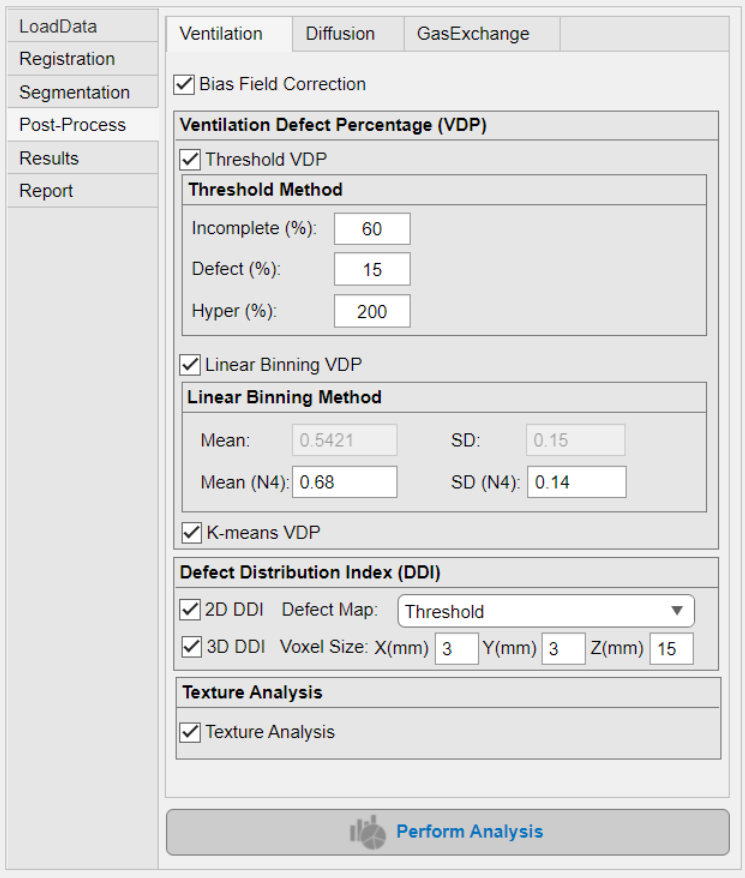
Figure S4. Interface and options of the Ventilation Post Process Panel. Ventilation defect percentage (VDP) and texture analysis can be performed on ventilation images. Users have the flexibility to adjust specific threshold values for the threshold method. In the linear binning method, mean and standard deviation parameters from a healthy reference can be modified. Defect distribution index can also be performed in 2D or 3D for a specific defect map. Texture analysis, utilizing the Gray Level Run Lengths Method (GLRLM) (1), is also integrated into the process. (GLRLM). For additional details, please refer to the user's manual document.


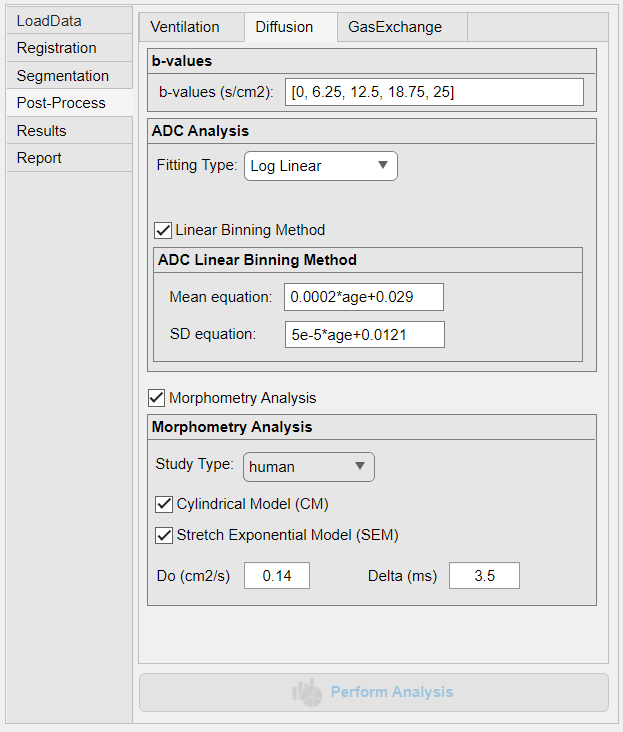
Figure S5. Interface and options of the Diffusion Post Process Panel. The diffusion analysis encompasses the generation of the apparent diffusion coefficient (ADC) and morphometry parameter maps (e.g., mean linear intercept, surface-to-volume ratio, and alveolar density, etc). ADC maps can be generated through log-linear fitting, weighted-linear fitting, non-linear fitting, or Bayesian methods. Morphometry analysis relies on cylindrical and stretch-exponential models. Additionally, linear binning analysis can be conducted using age-adjusted mean and standard deviation values from a healthy reference (equations can be modified by the user). For additional details, please refer to the user's manual document.


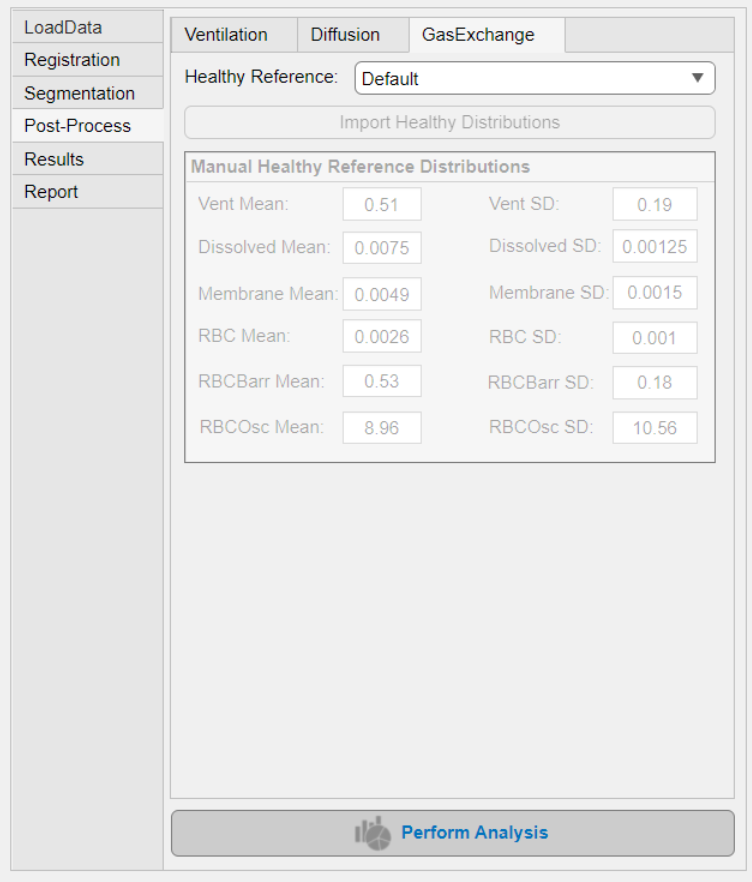


Figure S6. Interface and options of the Gas Exchange Post Process Panel. Three options in the dropdown menu for selecting the healthy reference distributions: default, import, and manual. Default will select the healthy distributions based on gas exchange data from Cincinnati’s Children’s Hospital Center. The user can also import a healthy distribution file in (.mat) format; otherwise, manual values are provided, and users have the option to modify these values as needed. For additional details, please refer to the user's manual document.


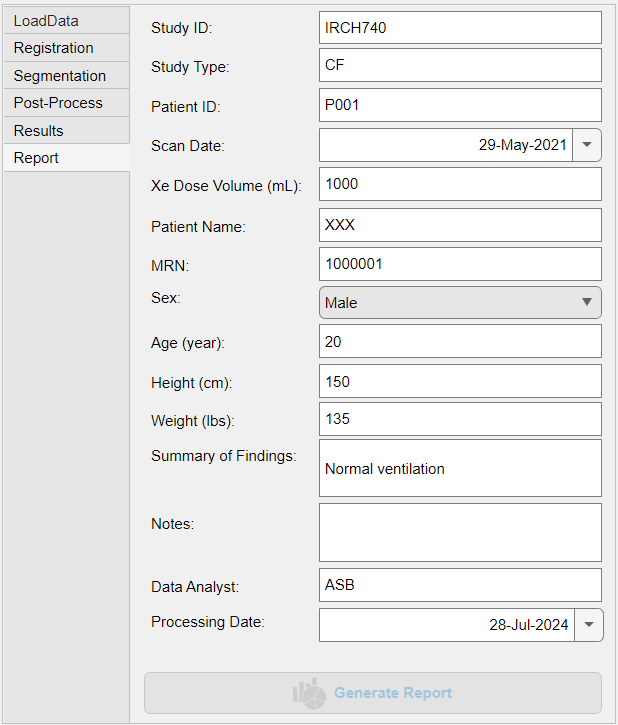


Figure S7. Interface and options of the Patient Report Panel. A PDF report will be automatically generated, incorporating information supplied by the data analyst (as illustrated in the figure) and the results of the image analysis.


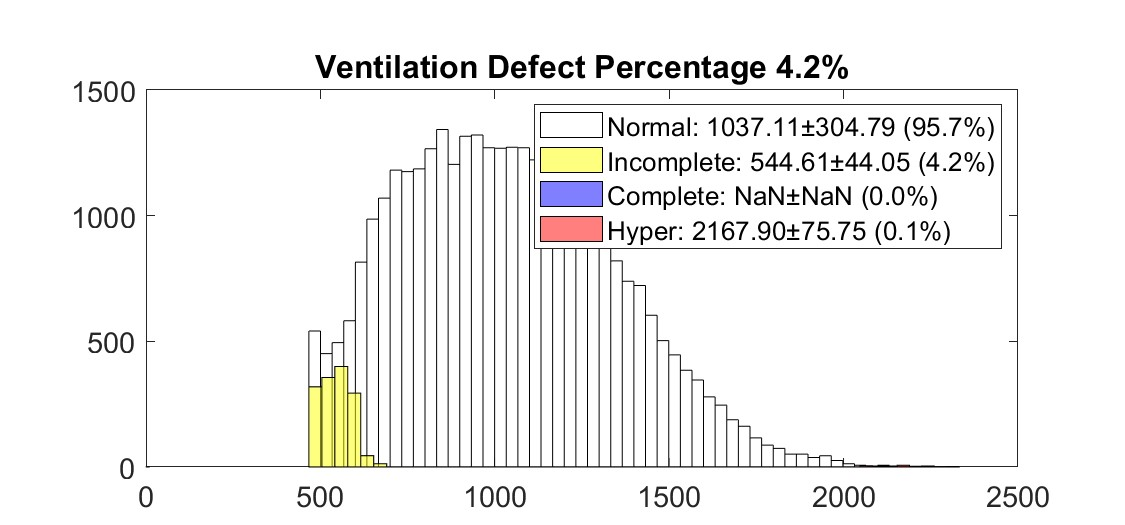


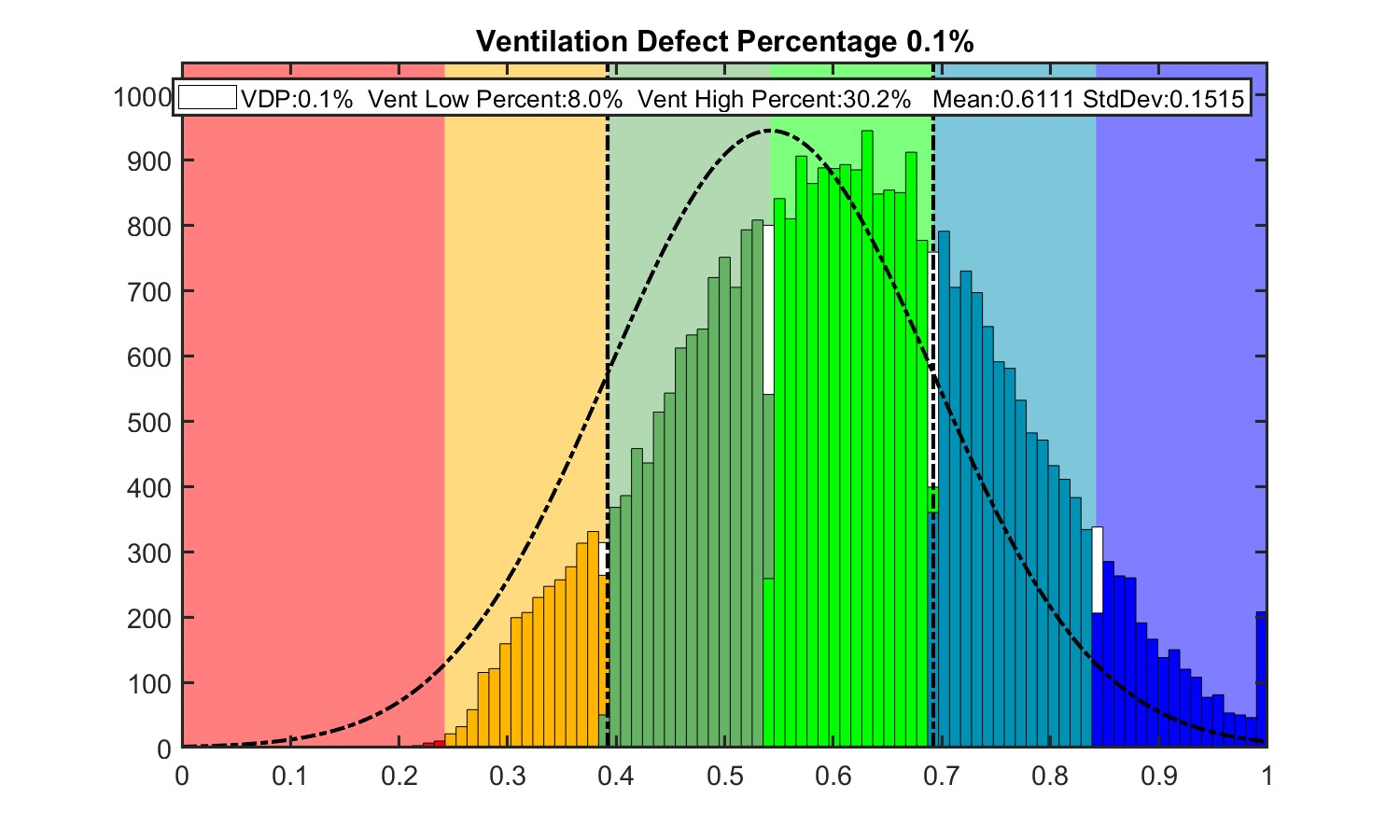
Figure S8-A. An example of a VDP histogram resulted from the VDP threshold method.

Figure S8-B. An example of a VDP histogram resulted from the VDP linear binning method.


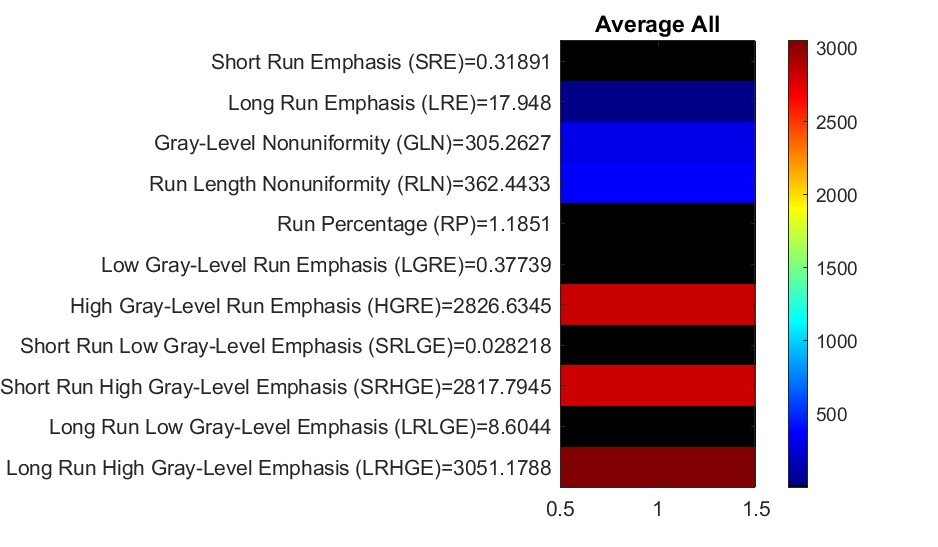


Figure S8-C. An example of mean parameters of gray level run lengths method (GLRLM) (1) analysis.

**
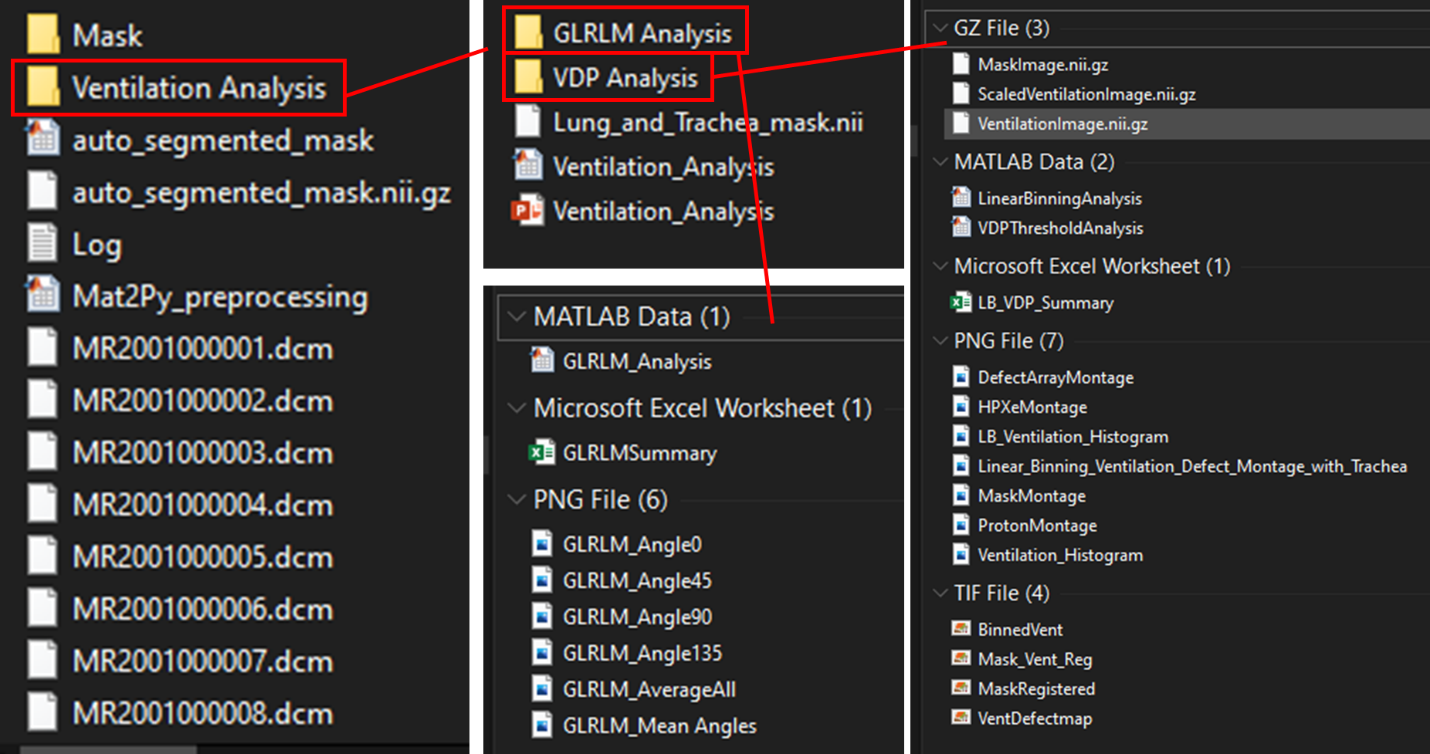
**

Figure S9: Ventilation analysis file output. The main analysis results will be saved in a folder named "Ventilation Analysis." Within this folder, two subfolders will be present, one for the VDP analysis and another for the texture analysis. Additionally, the main analysis output will be saved as a .mat file, while a report summarizing the findings will be saved as a .pptx file. This comprehensive file organization ensures easy access to the results and facilitates further examination and documentation of the ventilation analysis outcomes.


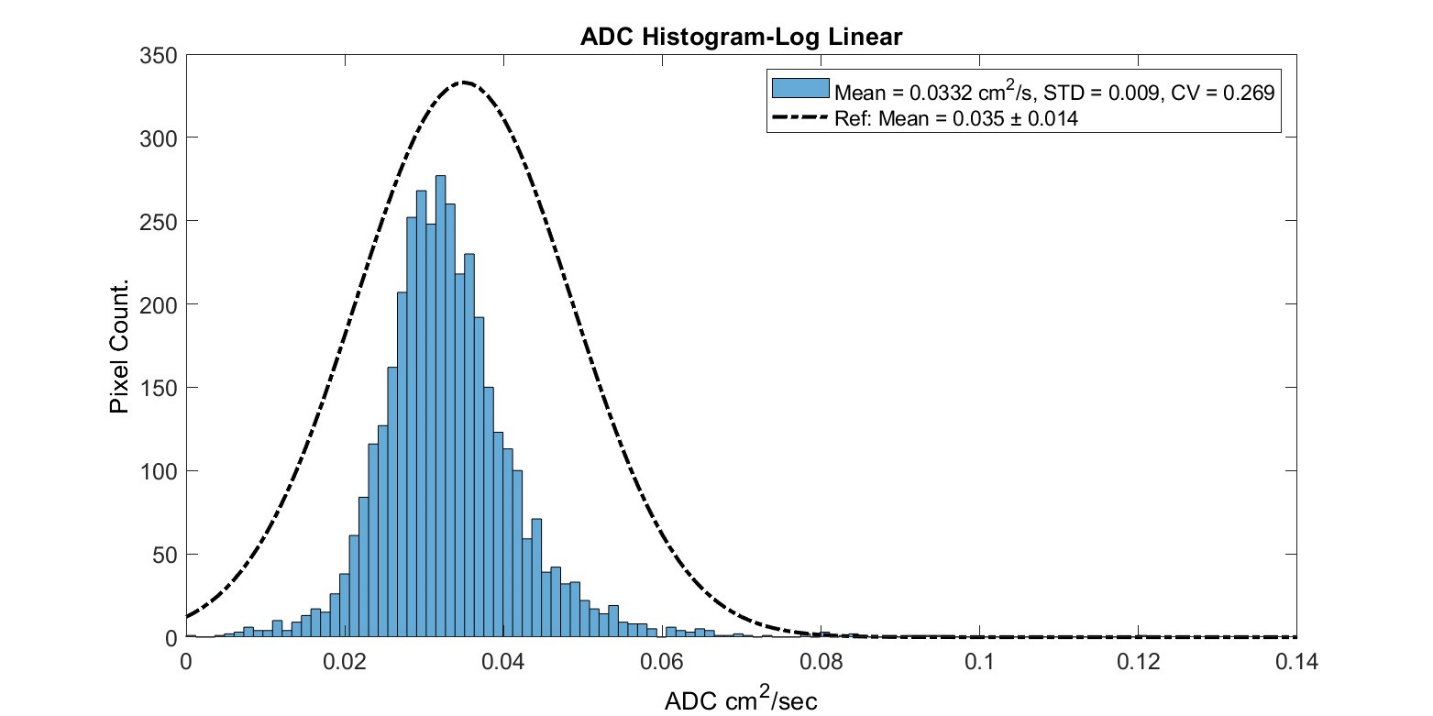
Figure S10-A. An example of an ADC histogram resulted from log-linear fitting method.


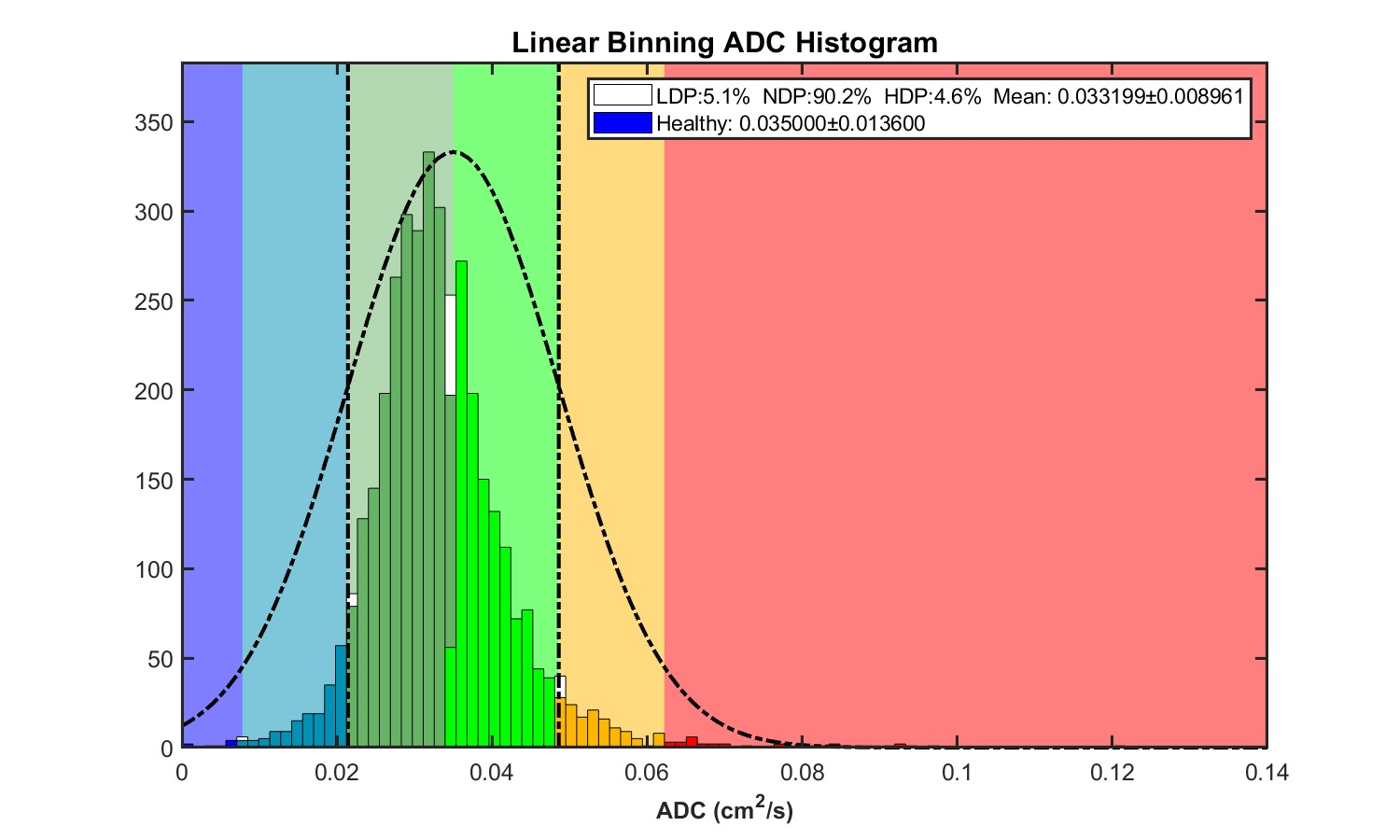


Figure S10-B. An example of an ADC histogram resulted from linear binning method.

**
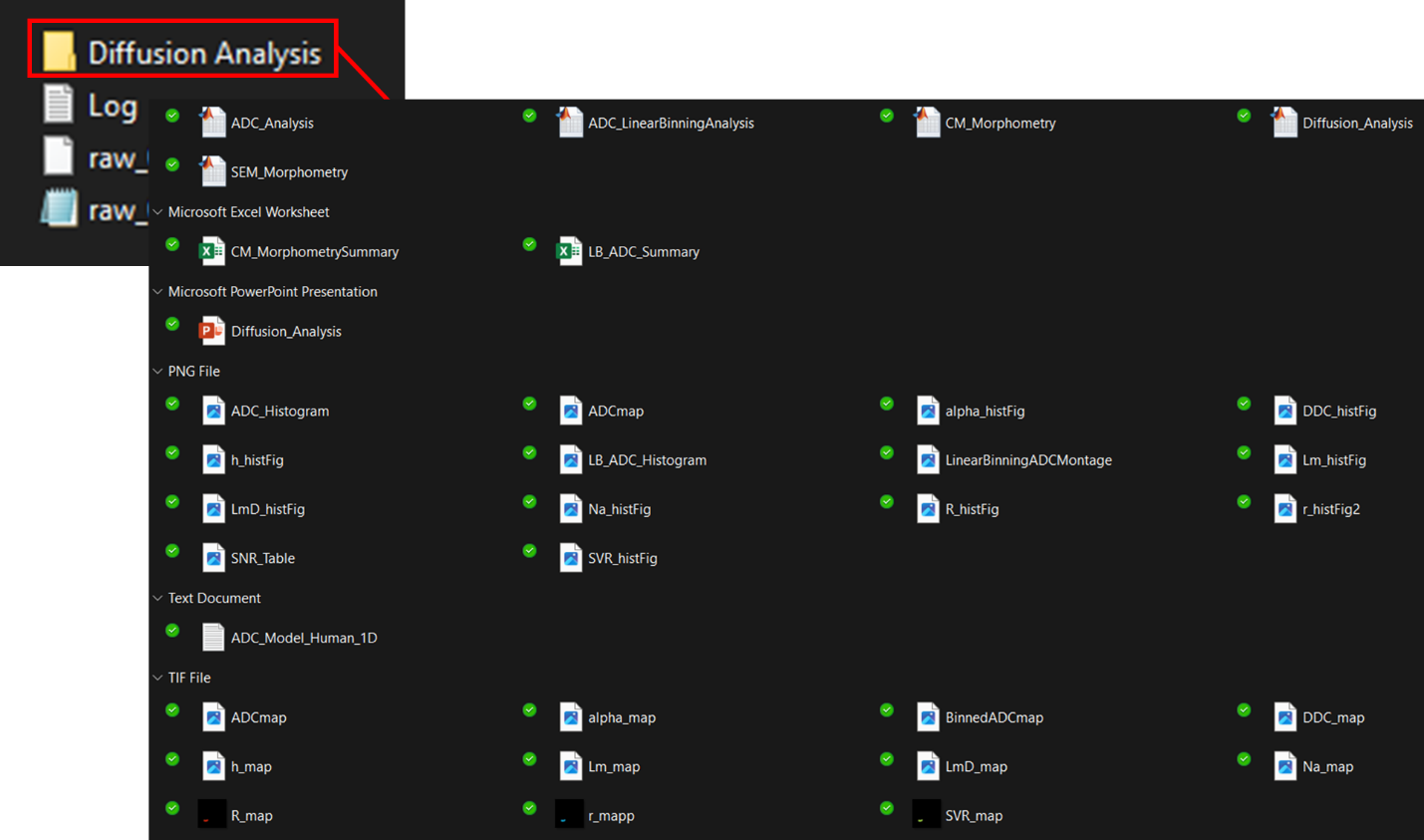
**

Figure S11: Diffusion analysis file output. The main analysis results will be saved in a folder named "Diffusion Analysis." The main analysis output will be saved as a .mat file, while a report summarizing the findings will be saved as a .pptx file.


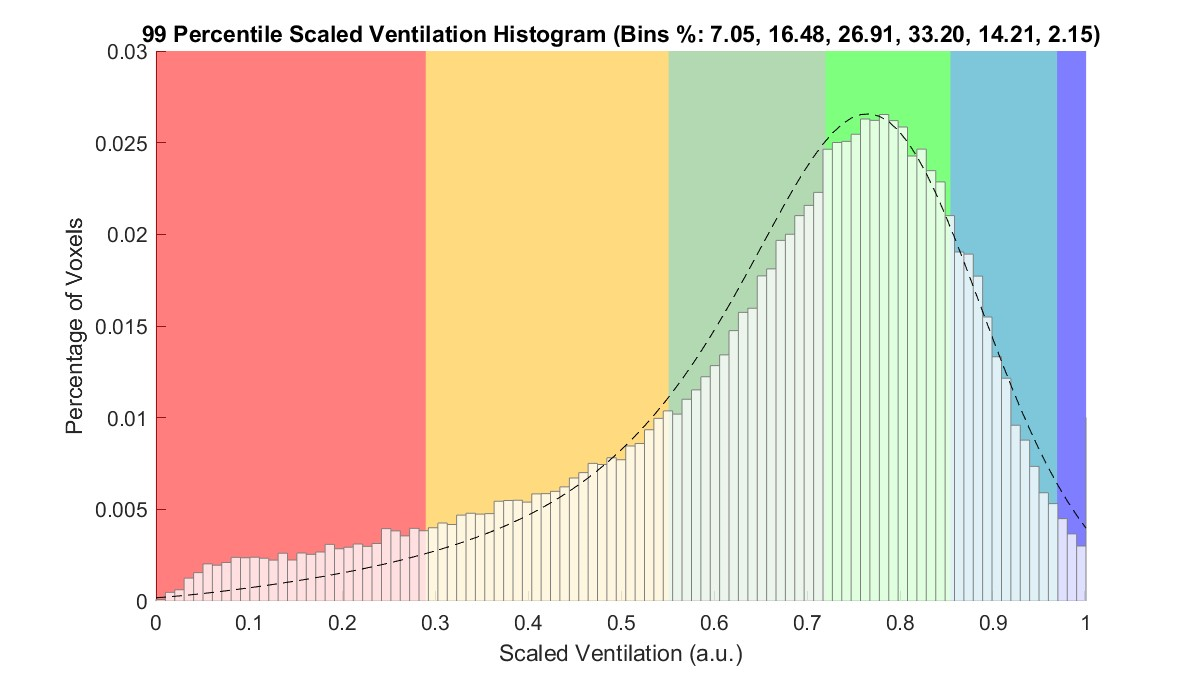
Figure S12-A. An example of gas-ventilation histogram resulted from linear binning method.


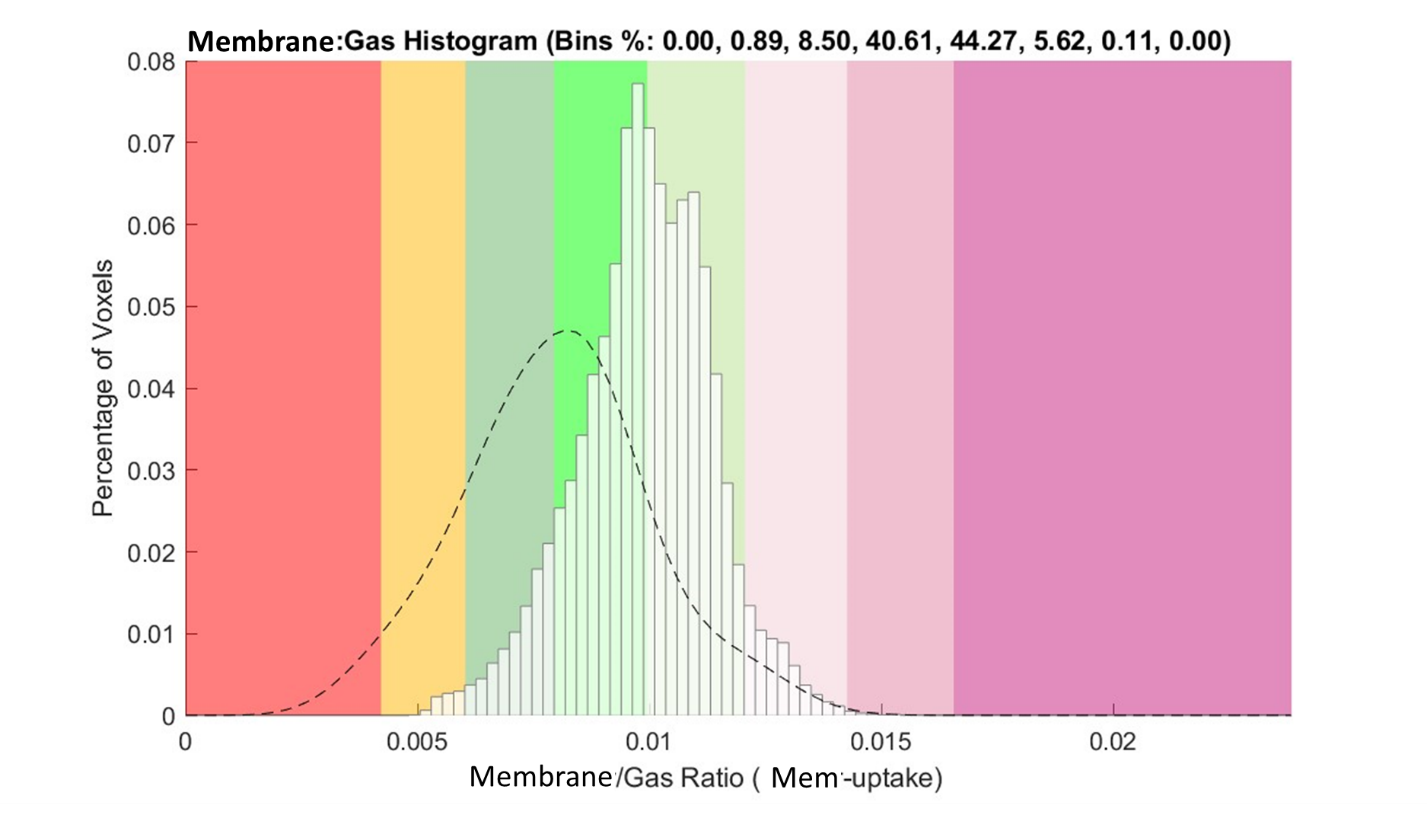


Figure S12-B. An example of membrane:Gas histogram resulted from linear binning method.


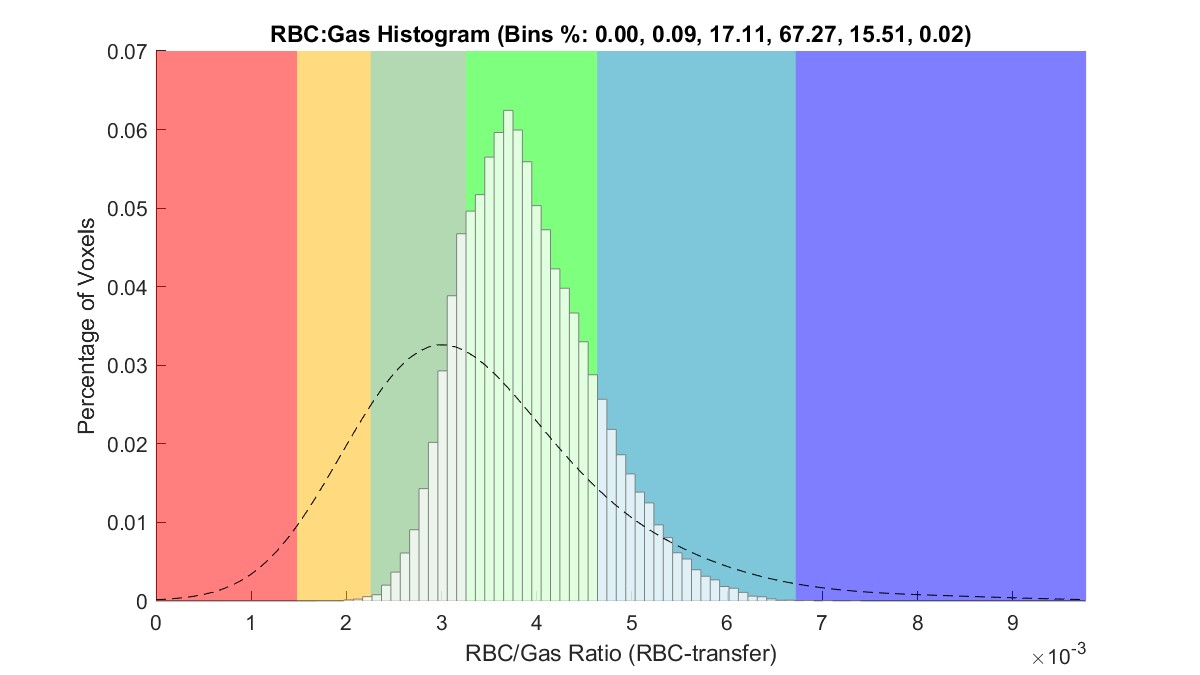
Figure S12-C. An example of RBC:Gas histogram resulted from linear binning method.


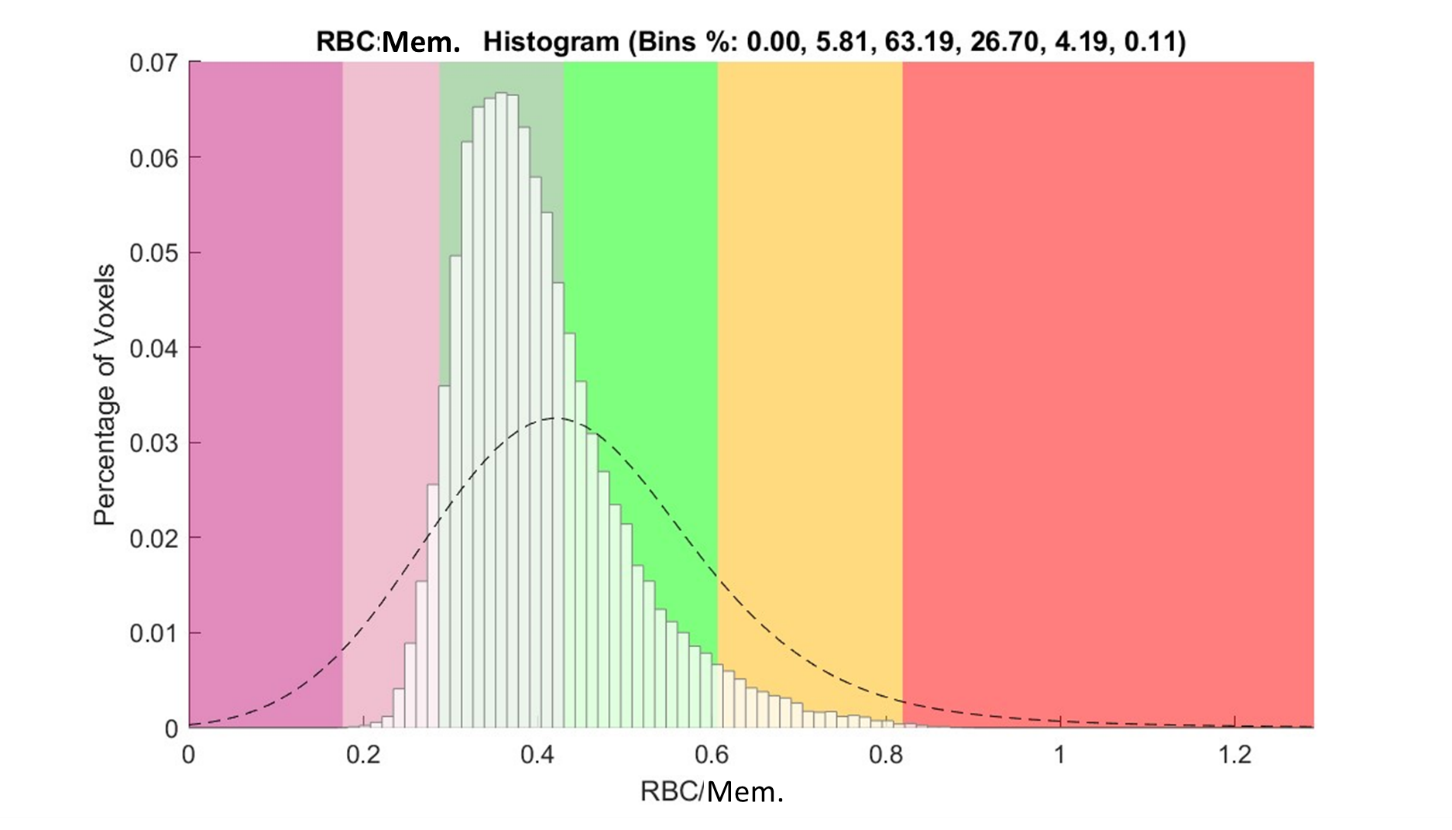


Figure S12-D. An example of RBC:Membrane histogram resulted from linear binning method.


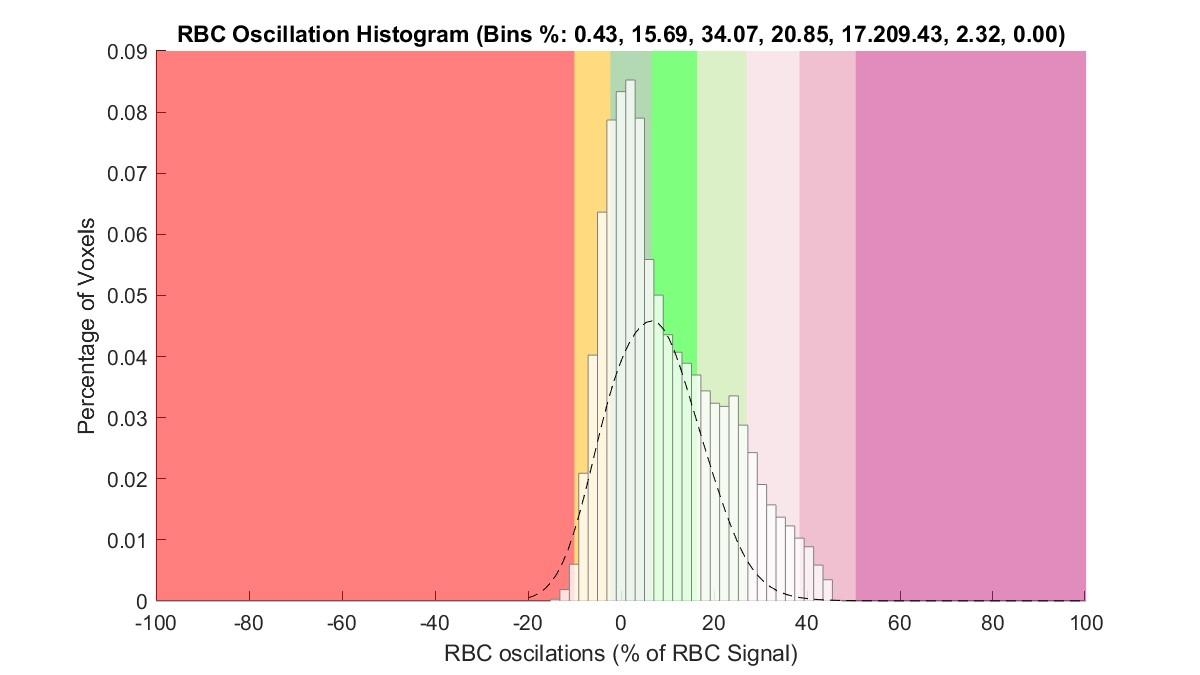
Figure S12-E. An example of RBC Oscillation histogram resulted from linear binning method.

**
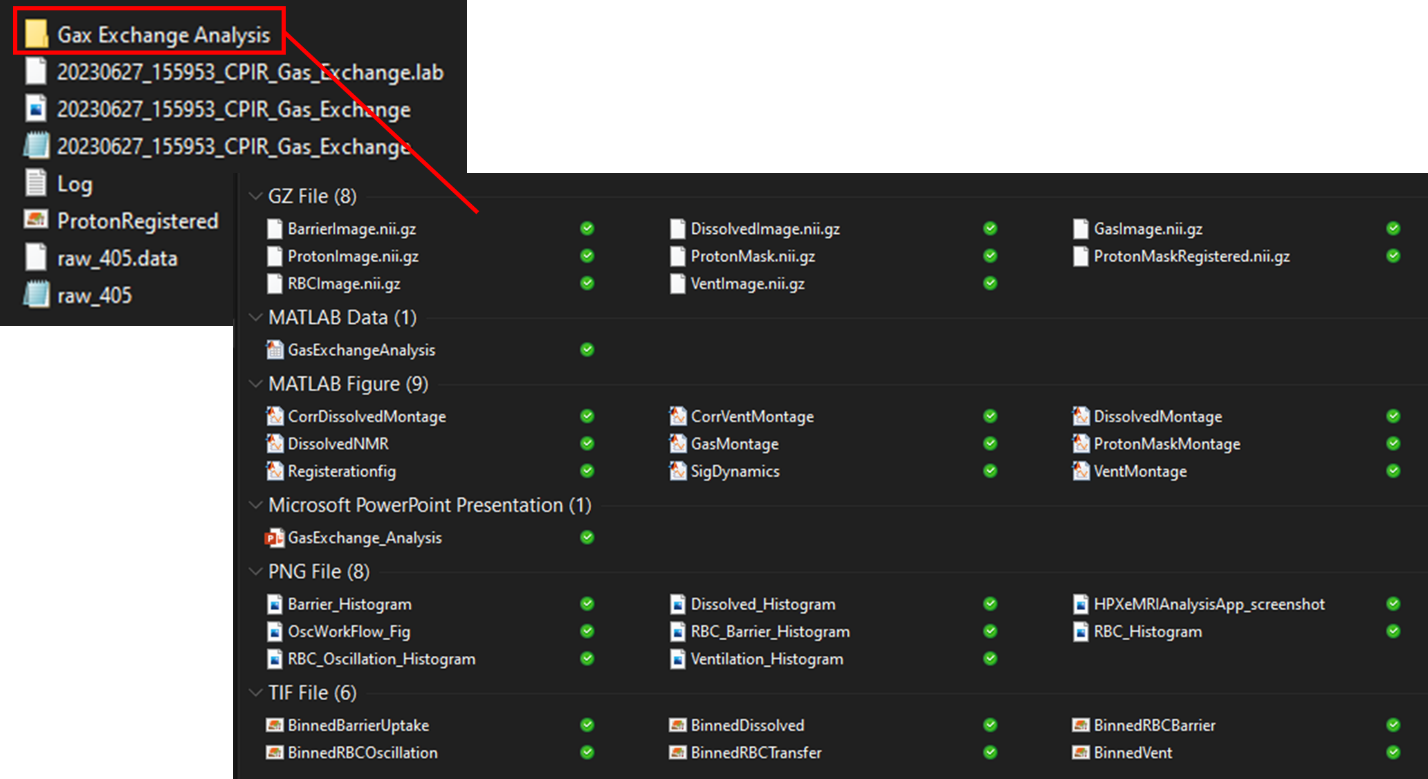
**

Figure S13: Gas Exchange analysis file output. The main analysis results will be saved in a folder named "Gas Exchange Analysis." The main analysis output will be saved as a .mat file, while a report summarizing the findings will be saved as a .pptx file.

**References**

1. Galloway MM. Texture analysis using gray level run lengths. Computer graphics and image processing 1975;4.2 (1975): 172-179.
